# Loss of MAX results in meiotic entry in mouse embryonic and germline stem cells

**DOI:** 10.1038/ncomms11056

**Published:** 2016-03-30

**Authors:** Ayumu Suzuki, Masataka Hirasaki, Tomoaki Hishida, Jun Wu, Daiji Okamura, Atsushi Ueda, Masazumi Nishimoto, Yutaka Nakachi, Yosuke Mizuno, Yasushi Okazaki, Yasuhisa Matsui, Juan Carlos Izpisua Belmonte, Akihiko Okuda

**Affiliations:** 1 Division of Developmental Biology, Research Center for Genomic Medicine, Saitama Medical University, Yamane Hidaka, Saitama 350-1241, Japan; 2 Gene Expression Laboratory, Salk Institute for Biological Studies, 10010 North Torrey Pines Road, La Jolla, California 92037, USA; 3 Universidad Católica San Antonio de Murcia (UCAM) Campus de los Jerónimos, No. 135, Guadalupe, 30107 Murcia, Spain; 4 Division of Translational Research, Research Center for Genomic Medicine, Saitama Medical University, Yamane Hidaka, Saitama 350-1241, Japan; 5 Division of Functional Genomics and Systems Medicine, Research Center for Genomic Medicine, Saitama Medical University, Yamane Hidaka, Saitama 350-1241, Japan; 6 Cell Resource Center for Biomedical Research, Institute of Development, Aging and Cancer, Tohoku University, Sendai 980-8575, Japan; 7 Japan Agency for Medical Research and Development and Development-Core Research for Evolutionary Science and Technology (AMED-CREST), Tokyo 100-0004, Japan

## Abstract

Meiosis is a unique process that allows the generation of reproductive cells. It remains largely unknown how meiosis is initiated in germ cells and why non-germline cells do not undergo meiosis. We previously demonstrated that knockdown of *Max* expression, a gene encoding a partner of MYC family proteins, strongly activates expression of germ cell-related genes in ESCs. Here we find that complete ablation of *Max* expression in ESCs results in profound cytological changes reminiscent of cells undergoing meiotic cell division. Furthermore, our analyses uncovers that *Max* expression is transiently attenuated in germ cells undergoing meiosis *in vivo* and its forced reduction induces meiosis-like cytological changes in cultured germline stem cells. Mechanistically, *Max* depletion alterations are, in part, due to impairment of the function of an atypical PRC1 complex (PRC1.6), in which MAX is one of the components. Our data highlight MAX as a new regulator of meiotic onset.

## Abstract (web summary)

The mechanisms that trigger meiosis in germ cells and halt this process in non-germline cells are unclear. Here, the authors show that knockout of *Max* in embryonic stem cells results in meiotic onset in a mechanism that involves the PRC1 complex.

## Graphical abstract

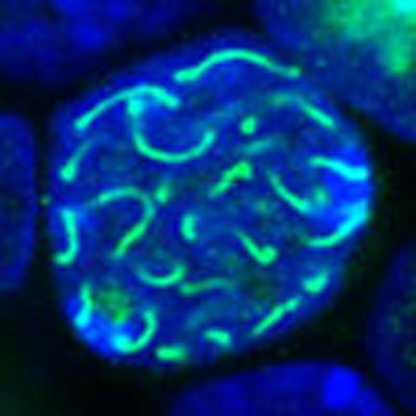

Meiosis is a specialized cell division process that produces gametes with a haploid genome^1^. Up to date, several reports have demonstrated significant conservation among species in the molecular basis underlying meiosis^2^. However, the molecular mechanisms that allow switching from mitotic to meiotic cell division in germ cells do not appear to be conserved across species or even between sexes within the same species. *Stra8*, whose expression is upregulated by retinoic acid (RA), has been shown to play crucial roles in premeiotic DNA replication^3^,^4^,^5^,^6^ and constitutes a physiological and crucial regulator of the initiation of meiosis in both sexes^7^,^8^,^9^,^10^. In addition to the RA–*Stra8* signalling pathway, it is plausible that unknown negative regulator of meiosis may prevent its premature entry in germ cells or ectopic induction in non-germ cells.

Recently, we demonstrated that knockdown of expression of the *Max* gene, encoding an indispensable partner for transcription factor c-MYC in embryonic stem cells (ESCs), leads to strong induction of germ cell-related gene expressions^11^. Notably, loss of *Max* expression in ESCs does not significantly alter the expression levels of primordial germ cell (PGC) specification genes such as *Blimp1* and *Stella* (also known as *Prdm1* and *Dppa3*, respectively), but rather selectively upregulates expression of genes related to meiotic cell division^11^. This preferential expression of meiosis-related genes in *Max* knockdown ESCs prompted us to explore the possibility that MAX might be part of the mechanism that safeguards meiosis by controlling the physiological timing of meiosis onset and preventing ectopic meiosis.

In this study, we found that *Max* depletion in ESCs not only upregulated the expression of meiosis-related genes but also induced the cytological changes reminiscent of germ cells at leptotene and zygotene stages of meiosis. Furthermore, our data revealed that these cytological changes even occurred in ESCs cultured in stringent 2i condition that renders ESCs refractory to cellular differentiation. This implies a direct conversion of *Max*-null ESCs to a meiosis-like state bypassing PGC differentiation. Moreover, we demonstrated that *Stra8*, but not *Blimp1*, was required for these cytological changes. Our analyses revealed that the *Max* gene undergoes a strong decline in expression during physiological meiosis in both male and female germs cells. Forced reduction of *Max* expression levels in germline stem cells (GSCs)^12^,^13^ by lentivirus-mediated knockdown induced meiosis-like cytological changes. Our findings in *Max*-null ESCs may reflect a physiological role for MAX during *in vivo* meiosis. *Max*-null ESCs may serve as a useful *in vitro* tool for studying the molecular mechanisms governing mitotic versus meiotic cell divisions. Mechanistically, our data indicate that these cytological changes are the result of loss of function of a variant PRC1 complex (PRC1.6) in which MAX is a component^14^,^15^.

## Results

### Induction of meiosis-related genes in *Max*-null ESCs

**Figure 1:**
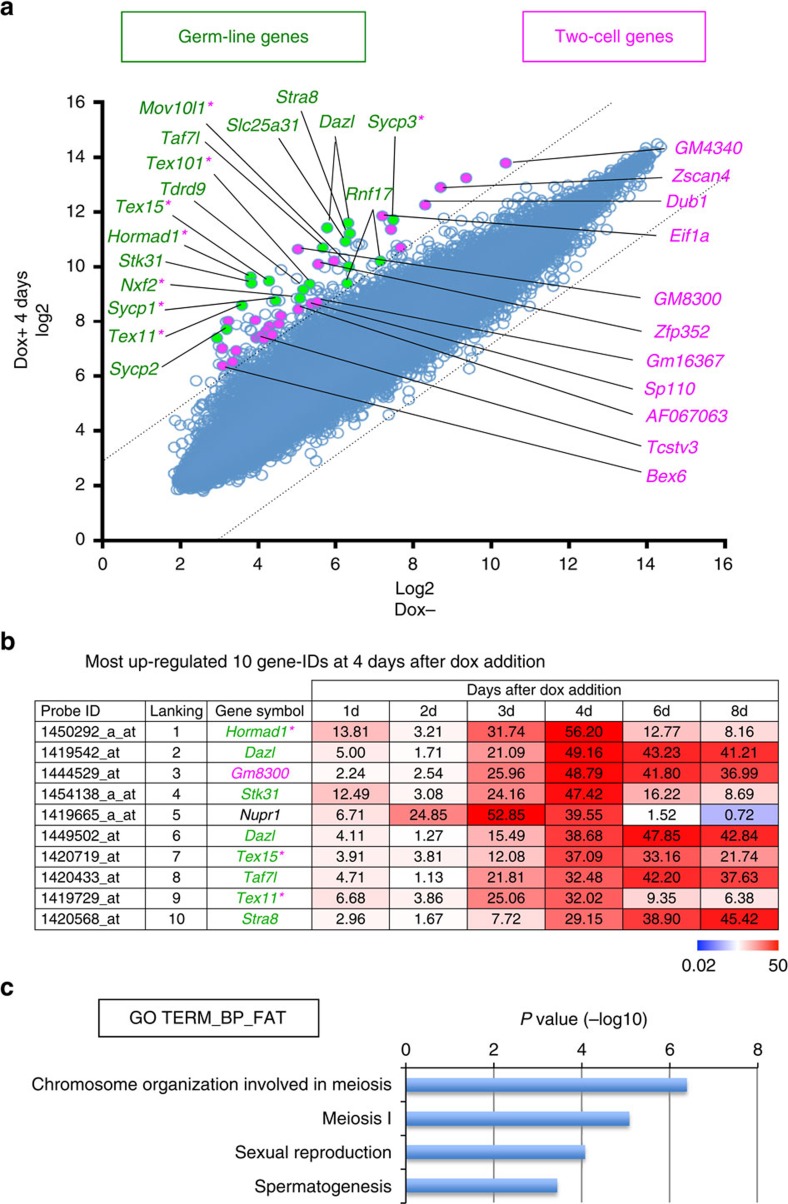
Elevation of germ cell- and two-cell embryo-related genes in ESCs subjected to *Max* expression ablation. (**a**) Scatter plots of DNA microarray data from untreated and Dox-treated *Max*-null ESCs for 4 days using our previously deposited DNA microarray data under accession number GSE27881. Among genes with highly elevated expression in Dox-treated *Max*-null ESCs compared with that in untreated control cells (eightfold or higher), genes related to germ cells (green) and/or two-cell embryos denoted by Macfarlan *et al.*^18^ (pink) are highlighted. Genes belonging to both categories are green with pink asterisks added to the gene names. (**b**) List of probe identifications (IDs) that showed the most conspicuous upregulation (top 10) in *Max*-null ESCs treated with Dox for 4 days (4d) and their expression changes during *Max* expression ablation in ESCs. *Dazl* is duplicated with different probe IDs. (**c**) GO analyses of genes showing more than eightfold higher expression in Dox-treated *Max*-null ESCs using DAVID software. The *P* value cutoff was set to <1 × 10^−3^.

Our previous RNA interference screen uncovered *Max* as a strong suppressor of the expression of germ cell-related genes such as *Ddx4* (also known as *Mvh*, mouse Vasa homologue) and *Dazl*, in ESCs^11^. Owing to residual *Max* expression in knockdown experiments, we hypothesized that *Max* knockout may elicit more profound effects. We first analysed our *Max*-null ESC transcriptome data set (GSE27881) reported previously. In these cells, the endogenous *Max* gene was homozygously disrupted, and doxycycline (Dox)-regulatable expression of MAX was introduced using the tetracycline-off system^16^. Consistent with our hypothesis, expression levels of many germ cell-related genes were elevated in *Max*-null ESCs, of which most are meiosis-related (Fig. 1a,b and Supplementary Fig. 1). Some of the meiotic genes, such as *Stra8* and *Dazl*, showed progressive increase in the expression levels following Dox treatment, while expression of others, such as *Hormad1* and *Stk31*, peaked at around day 4 and abruptly dropped thereafter. However, it is unknown whether the latter expression dynamics are caused by a negative feedback loop of regulation of these meiotic genes or simply a consequence of extensive cell death phenotype associated with meiotic-like *Max*-null ESCs. Unlike meiosis-related genes, PGC specification genes such as *Blimp1* and *Dppa3* did not show appreciable alterations in their expression levels on Dox treatment. We also noted that the expression levels of genes encoding meiosis-specific cohesion components, such as *Rec8* and *Smc1b*, were elevated, to a lesser extent, in Dox-treated cells compared with untreated controls. We also examined the expression of genes encoding key regulators of DNA recombination and found most of them remained unchanged, although a modest increase was evident in *Spo11* expression. These results implied that *Max*-null ESCs exhibited a partial, but not complete, gene expression pattern characteristic of meiotic cells (Supplementary Fig. 1). In addition, we also observed increased expression of two-cell embryo signature genes such as *Zscan4* and *Dub1* (refs 17, 18; Fig. 1a,b and Supplementary Fig. 1). However, flow cytometric analyses of Dox-inducible *Max*-null ESCs bearing a DsRed reporter gene, which faithfully recapitulates the endogenous *Zscan4* expression profile with an aid of 5′-flanking region of the gene, indicated that DsRed- and STRA8-positive cells, both of which became prominent after *Max* depletion in ESCs, did not significantly overlap, but were rather mutually exclusive (Supplementary Fig. 2). These data implied that meiosis-like induction and activation of two-cell embryo gene signatures are independent phenomena. As expected, gene ontology (GO) analysis revealed over-representation of genes related to meiosis and sexual reproduction (Fig. 1c).

### *Max*-null ESCs show meiosis-like cytological changes

**Figure 2:**
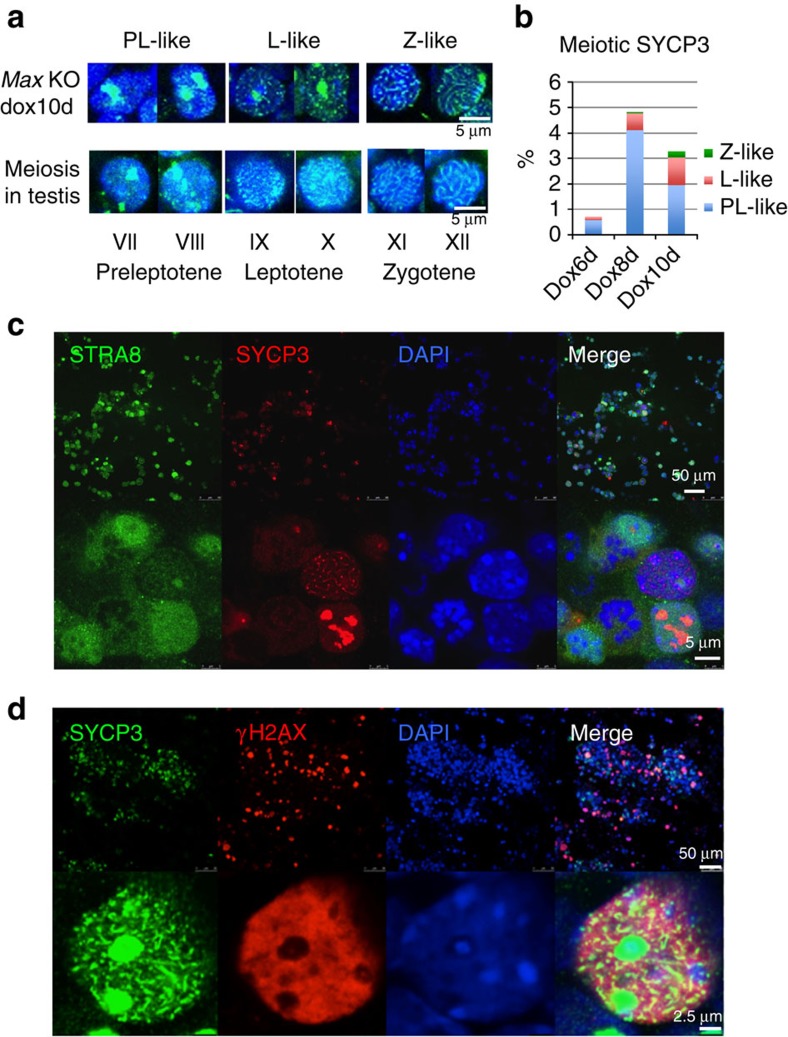
Cytological changes reminiscent of cells undergoing meiotic cell division processes. (**a**) Meiosis-like SYCP3-staining patterns in *Max*-null ESCs treated with Dox for 10 days. The meiosis-like SYCP3-staining patterns in *Max*-null ESCs were classified as preleptotene (PL)-, leptotene (L)- and zygotene (Z)-like patterns according to the criteria shown in Supplementary Fig. 3. (**b**) Frequency of preleptotene-, leptotene- and zygotene-like SYCP3-staining patterns in *Max*-null ESCs at the indicated days after Dox administration. (**c**) Co-immunostaining analyses of *Max*-null ESCs treated with Dox for 9 days with antibodies against SYCP3 and STRA8. (**d**) *Max*-null ESCs treated with Dox for 10 days were co-immunostained for SYCP3 and γH2A.X.

Expression of meiosis-related genes led us to examine cytological changes in *Max*-null ESCs. To this end we performed immunocytochemical analyses with an antibody against SYCP3, one of the major components of the synaptonemal complex^19^,^20^. After 8 days of Dox treatment, about 4–5% of *Max*-null ESCs showed a SYCP3-immunostaining pattern that is reminiscent of germ cells undergoing meiosis (Fig. 2a,b). We observed about 20% STRA8-positive cells among all *Max*-null ESCs treated with Dox for 6 days (Supplementary Fig. 2). Most STRA8-positive cells, however, failed to proceed to the stage acquiring a meiosis-like SYCP3-staining pattern. To assess the stages of meiosis in meiosis-like cells from *Max*-null ESCs, we established a criterion to distinguish the stages based on the average length of SYCP3-stained regions observed in the chromosomes of germ cells in seminiferous tubules of the testis (Supplementary Fig. 3). Our analyses indicated that meiosis-like cells derived from *Max*-null ESCs following 6 days' Dox treatment were mostly preleptotene-like, with some cells progressed to the leptotene-like state. And longer Dox treatment (8 or 10 days) increased the percentages of these cells and also yielded cells bearing SYCP3-staining patterns similar to germ cells at zygotene stages of meiotic cell division (Fig. 2b). However, no cell was scored as pachytene-like even with extended Dox treatment (Fig. 2a,b). To confirm these observations, we conducted co-staining with antibodies against SYCP3 together with other meiotic markers. First, similar to the normal progression of meiosis, we found that STRA8 expression was more enriched in cells representing the early stages of meiosis and much less in cells at a more advanced stage with a zygotene-like SYCP3-staining pattern (Fig. 2c). We also found that cells with a leptotene-like SYCP3-staining pattern showed a strong γH2AX signal, similar to meiotic cells at this stage (Fig. 2d). Furthermore, REC8 and SYCP3 were co-localized in some, but not all, of the cells with a zygotene-like SYCP3-staining pattern (Supplementary Fig. 4a), likely due to less conspicuous induction of *Rec8* compared with that of other meiotic marker genes such as *Stra8* and *Sycp3* (Supplementary Fig. 1). We also conducted immunostaining with an antibody against SYCP1 that is known to play a crucial role in the formation of the synaptonemal complex at the pachytene stage of meiosis, in which two paired homologous chromosomes (four chromosomes in total) are brought into a juxtaposition as a prerequisite step for the subsequent crossover^21^,^22^,^23^. Although SYCP1 expression was detected in some cells, we did not observe extensive overlap of SYCP1 signals with that of the SYCP3 on chromosomes (Supplementary Fig. 4b). These results were consistent with the data shown in Fig. 2b and imply that *Max* deficiency arrests ESCs at the crux of the pachytene stage of meiosis-like processes. Because aurora kinase is a known critical regulator of meiosis^24^, we examined changes in the expression of aurora kinase A, B and C in *Max*-null ESCs by western blot analyses. All three aurora kinases exhibited reductions in their protein expression levels, although the reduction of aurora kinase C was marginal (Supplementary Fig. 5a). Next, we examined the effect of a pan-aurora kinase inhibitor, VX680, and found that it did not noticeably alter the magnitude of meiosis-like changes in *Max*-null ESCs (Supplementary Fig. 5b,c). Because aurora kinase C is the only aurora kinase with an unequivocal contribution to meiosis among the three aurora kinases and localization of aurora kinase C at chromocentres becomes apparent at the diplotene stage^24^, no apparent effect of the aurora kinase inhibitor may be reasonable in our system in which the meiotic process was arrested at or before the pachytene stage (Fig. 2). Next, we examined whether loss of *Max* expression in ESCs was accompanied by alterations in genomic DNA methylation levels. To this end we compared the levels of 5-methyl cytosine (5mC) and 5-hydroxymetyl cytosine (5hmC) between Dox-untreated and -treated ESCs for 8 days. Although 5mC levels remained rather constant on *Max* depletion, our analyses demonstrated an elevation in the levels of 5hmC by about twofold in Dox-treated *Max*-null ESCs (Supplementary Fig. 6a). We also examined the methylation status of imprinting genes because erasure of imprinting is one of the hallmarks of germ cell development. We found that differentially methylated regions (DMRs) of maternally methylated *Snrpn* and *Igf2r* genes were almost completely demethylated in *Max* expression-ablated ESCs, implying that activation of the meiotic programme in these cells was coupled with imprinting erasure (Supplementary Fig. 6b,c). We also found similar, but less significant, induction of demethylation in one of the DMRs in the paternally methylated *H19* gene (Supplementary Fig. 6d). The almost complete methylation of DMRs in the *H19* gene of Dox-untreated ESCs reflects the usual DNA methylation status of this locus in mouse ESCs cultured in conventional mouse ESC medium^25^,^26^,^27^.

### Vitamin C enhances meiosis-related changes in *Max*-null ESCs

**Figure 3:**
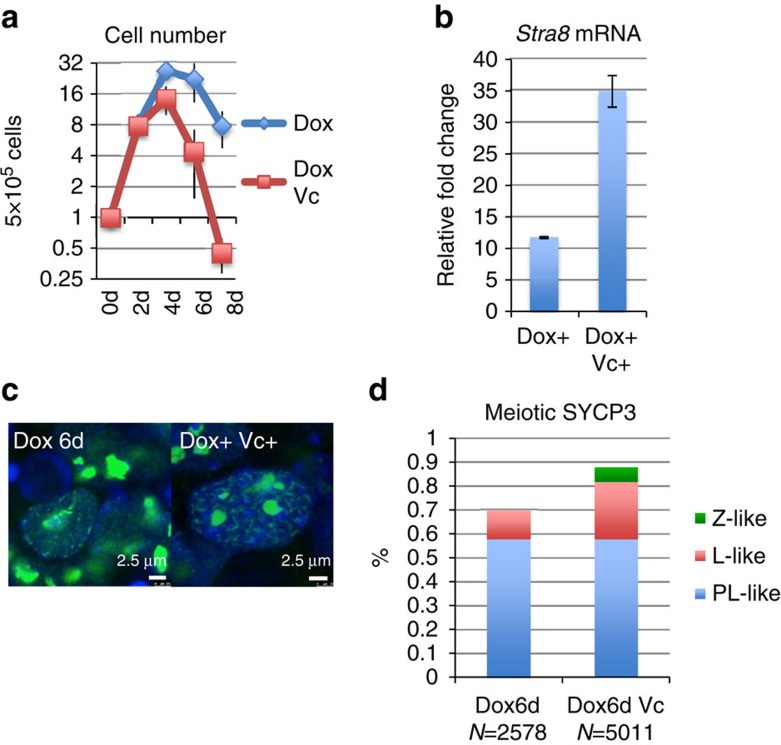
Vitamin C enhances meiosis-related changes in *Max* expression-ablated ESCs. (**a**) Total number of viable Dox-treated *Max*-null ESCs cultured in the presence or absence of vitamin C at the indicated days after Dox addition. The number of cells at day 0 (0d) was arbitrarily set to one (*n*=3, means±s.d.). (**b**) Expression levels of the *Stra8* gene in Dox-treated *Max*-null ESCs cultured in the presence or absence of vitamin C for 4 days (4d). The expression level of the *Stra8* gene in untreated control *Max*-null ESCs was arbitrarily set to one (*n*=3, means±s.d.). (**c**) Representative meiosis-like SYCP3-staining patterns in Dox-treated *Max*-null ESCs cultured in the absence or presence of vitamin C for 6 days (6d). (**d**) Frequency of meiosis-like SYCP3-staining patterns in *Max*-null ESCs treated only with Dox for 6 days or together with vitamin C.

Since apoptosis is a prominent phenotype of *Max*-null ESCs, we investigated whether apoptosis might be triggered by meiosis-like changes or elevated levels of reactive oxygen species in *Max*-null ESCs^16^. Towards this end we treated *Max*-null ESCs with vitamin C, a compound that has been described both as an antioxidant and as a potentiating factor in meiosis through activation of ten-eleven translocation 1 (Tet1)^28^,^29^. Vitamin C treatment of *Max*-null ESCs did not reduce but instead significantly accelerated cell apoptosis (Fig. 3a). This was accompanied by elevated *Stra8* expression (Fig. 3b). Vitamin C treatment also increased the frequency of meiosis-like SYCP3-staining patterns and yielded cells representing more advanced stages of meiosis compared with Dox treatment alone (Fig. 3c,d). These results implied that the meiosis-related changes rather than reactive oxygen species in *Max*-null ESCs are closely linked to apoptosis. To further explore the relationship between the meiosis-like changes and apoptotic cell death in *Max*-null ESCs, we suppressed the levels of apoptosis by treatment with the caspase inhibitor Z-VAD-FMK. We found that Z-VAD-FMK treatment increased the frequency of cells with meiosis-like changes, especially cells with preleptotene- and leptotene-like SYCP3-staining patterns (Supplementary Fig. 7). These observations implied that a substantial portion of meiotic-like *Max*-null ESCs were eliminated at rather early stages before proceeding to more advanced stages of meiosis. Apoptosis occurred likely as a result of the ESC culture condition, which is different from the niche environment found within the seminiferous tubules of the testis and the female genital ridge.

### Meiosis-like changes in *Max*-null ESCs depend on *Stra8* gene

**Figure 4:**
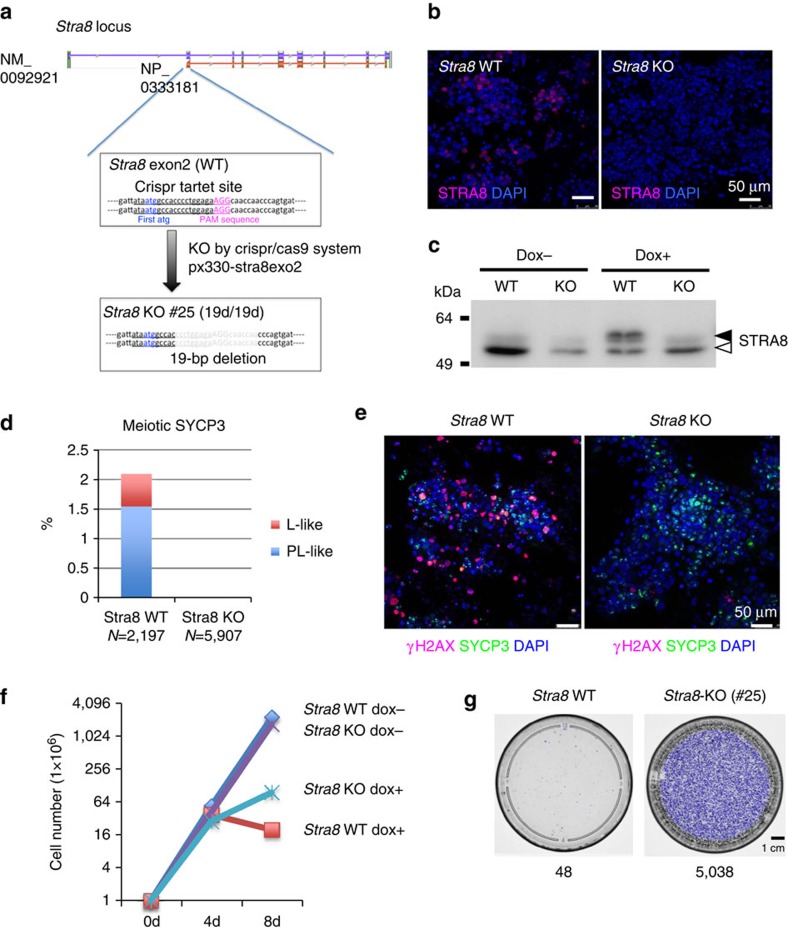
Induction of the *Max* expression ablation-mediated meiosis-like SYCP3-staining pattern in ESCs depends on the presence of a functional *Stra8* gene. (**a**) Schematic representation of CRISPR-Cas9-mediated disruption of the *Stra8* gene. Genomic analyses revealed that *Stra8* KO #25 clone had an out-of-frame mutation in both loci of the *Stra8* gene due to homozygous deletion of 19 base pairs located immediately downstream of the ATG initiation codon in exon 2 of the gene. (**b**) Confirmation of the absence of functional *Stra8* genes in the *Stra8* KO #25 *Max*-null ESC clone. The *Stra8* KO #25 clone and parental *Max*-null ESCs were treated with Dox for 9 days and then subjected to immunocytochemical analyses with an antibody against STRA8. (**c**) Western blot analyses of STRA8 in *Stra8* KO #25 clone and parental *Max*-null ESCs cultured in the presence or absence of Dox. Solid and open arrowheads indicate specific and non-specific bands, respectively. (**d**) Frequency of preleptotene-, leptotene- and zygotene-like staining patterns in the *Stra8* KO #25 clone and parental *Max*-null ESCs treated with Dox for 9 days. (**e**) Immunostaining analyses of γH2AX and SYCP3 in the Dox-treated (9 days) *Stra8* KO #25 clone and parental *Max*-null ESCs. (**f**) Increase in the recovery of viable Dox-treated *Max*-null ESCs due to homozygous knockout of the *Stra8* gene. The number of cells at day 0 was arbitrarily set to one. (**g**) *Max*-null ESCs (1 × 10^5^) with or without functional *Stra8* genes were individually transferred to 10-cm dishes and treated with Dox for 12 days. The cells were then subjected to Leishman's staining. Numbers under the dishes are the number of stained cell colonies. d, day; KO, knockout; WT, wild type.

To explore the relationship between the induction of *Stra8* gene expression and the meiosis-like SYCP3-staining patterns in our system, we knocked out the *Stra8* gene in *Max*-null ESCs using the CRISPR-Cas9 system^30^ (Fig. 4a). Immunocytochemical (Fig. 4b) and western blot (Fig. 4c) analyses confirmed that *Max* depletion-mediated induction of *Stra8* expression did not occur in *Stra8*-knockout ESCs. More importantly, loss of *Stra8* gene functions resulted in marked suppression of incidences of meiosis-like SYCP3-staining patterns in Dox-treated *Max*-null ESCs (Fig. 4d). We also found that disruption of the *Stra8* gene led to a significant reduction in γH2AX signals, a characteristic feature observed strongly at leptotene stages of meiosis (Fig. 4e). These data implied that, similar to normal meiotic cell division, expression of *Stra8* is indispensable for induction of meiotic-like patterns in *Max*-null ESCs^3^,^4^,^5^,^6^. It is also worth noting that *Stra8* knockout not only impaired the cytological changes reminiscent of meiosis but also significantly alleviated apoptotic cell death phenotype associated with *Max* knockout (Fig. 4f,g). Therefore, these results further suggest that the extensive cell death observed in *Max*-null ESCs is, at least in part, due to induction of meiosis.

### Meiosis-like changes in *Max*-null ESCs under 2i condition

**Figure 5:**
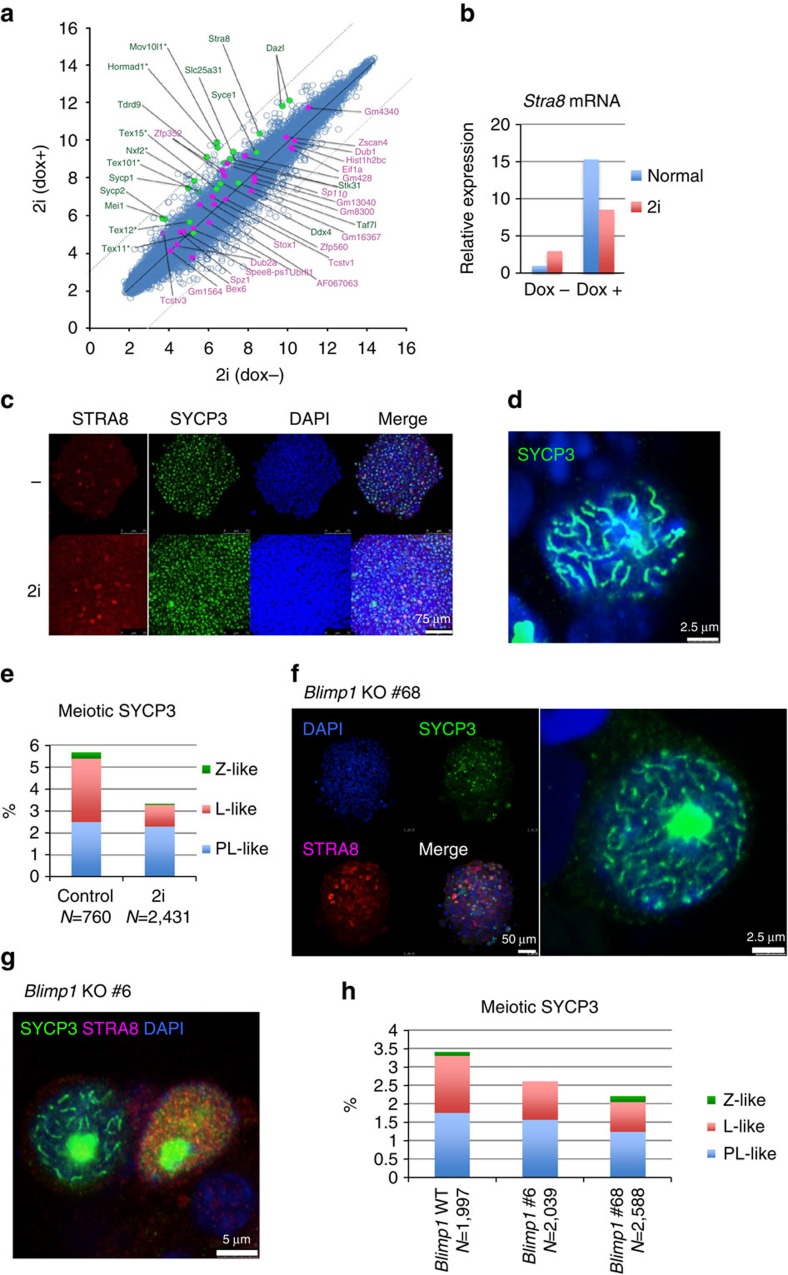
Occurrence of meiosis-like changes in *Max* expression-ablated ESCs without passing through the PGC state. (**a**) Scatter plots of DNA microarray data from untreated and Dox-treated *Max*-null ESCs cultured under the 2i+LIF condition. Genes highlighted in Fig. 1a are indicated by the same colours (green: germ cell-specific genes; pink: two-cell embryo-specific genes; green with an asterisk: genes belonging to both categories). (**b**) Measurement of *Stra8* mRNA levels by quantitative PCR in Dox-untreated and -treated *Max*-null ESCs cultured under conventional mouse ESC or 2i+LIF culture conditions. (**c**) Immunocytochemical analyses of STRA8 and SYCP3 in Dox-treated *Max*-null ESCs cultured under conventional mouse ESC or 2i+LIF culture conditions. (**d**) Representative image of the zygotene-like SYCP3-staining pattern observed in Dox-treated *Max*-null ESCs cultured under the 2i+LIF condition. (**e**) Frequency of meiosis-like SYCP3-staining patterns in Dox-treated *Max*-null ESCs cultured under conventional mouse ESC or 2i+LIF culture conditions. (**f**) Immunocytochemical analyses of STRA8 and SYCP3 in the Dox-treated *Blimp1* KO #68 *Max*-null ESC clone. Right panel shows a representative zygotene-like SYCP3-staining pattern. In this mutant clone, a single adenine nucleotide was inserted into both loci of exon 3 in the *Blimp1* gene, causing an out-of-frame mutation (for details, see Supplementary Fig. 8). (**g**) Leptotene-like immunostaining pattern of SYCP3 with high STRA8 expression (right) and the zygotene-like SYCP3-staining pattern (left) with low STRA8 expression in the Dox-treated *Blimp1* KO #6 *Max*-null ESC clone in which a large deletion had occurred in the region encompassing exon 3 and intron 3 in both loci of the gene (for details, see Supplementary Fig. 8). (**h**) Frequency of meiosis-like SYCP3-staining patterns in Dox-treated *Max*-null ESCs of *Blimp1* KO #68 and #6 clones. KO, knockout.

We have previously demonstrated that *Max*-null ESCs in the 2i+leukaemia inhibitory factor (LIF) condition with two kinase inhibitors against MEK and GSK3β (ref. 31), but not in conventional ESC medium, maintain normal expression levels of pluripotency markers such as OCT3/4, SOX2 and NANOG, and are able to self-renew indefinitely^16^. In this context, we sought to determine whether *Max* depletion leads to the activation of germ cell-related genes in ESCs even if the vast majority of cells maintain their pluripotent properties. First, DNA microarray analyses showed that the expression levels of germ cell-related genes were elevated even in Dox-treated *Max*-null ESCs under the 2i+LIF condition, although the induction levels were not as conspicuous as those observed in *Max*-null ESCs under conventional LIF+serum conditions (Fig. 5a). Quantitative PCR confirmed the induction of *Stra8* gene expression in both conditions (Fig. 5b). These results prompted us to investigate whether the cytological changes reminiscent of meiotic cell division also occur in Dox-treated *Max*-null ESCs under the 2i+LIF condition. We found that accumulation of STRA8 protein as well as meiosis-like SYCP3 staining became evident on loss of *Max* expression in ESCs under the 2i+LIF condition (Fig. 5c,d), although the frequency of these cytological changes was about half of that observed in conventional ESC culture conditions (Fig. 5e).

### Implications of direct onset of meiosis in *Max*-null ESCs

It has been shown that the 2i+LIF condition renders ESCs refractory to cellular differentiation^32^. The changes in meiosis reported in *Max*-null ESCs, even in the 2i+LIF condition, indicate that this induction occurs without passing through a PGC state that first becomes evident at around 7.0 days post coitum (dpc; early gastrulation stage) in mouse embryos^33^. This is supported by the fact that *Max* ablation in ESCs led to elevated expression levels of late PGC and meiosis-related genes, but did not elicit prominent induction of PGC specification genes. To further address this issue, and taking into account the fact that *Blimp1* is known to play crucial roles in the induction and maintenance of PGCs^34^,^35^, we knocked out the *Blimp1* gene in *Max*-null ESCs using the CRISPR-Cas9 system. After homozygous knockout of the *Blimp1* gene (Supplementary Fig. 8), the ESCs were treated with Dox to suppress *Max* expression for 9 days and then subjected to co-immunocytochemical analyses with antibodies against SYCP3 and STRA8. Strong induction of STRA8 as well as meiosis-like SYCP3-staining patterns became evident in the ESCs after loss of *Max* expression, even without a functional *Blimp1* gene (Fig. 5f–h), indicating that *Max*-null ESCs bypassed the PGC state and directly acquired features reminiscent of meiosis I phase.

### Meiosis-related changes in ESCs depend on retinoid

**Figure 6:**
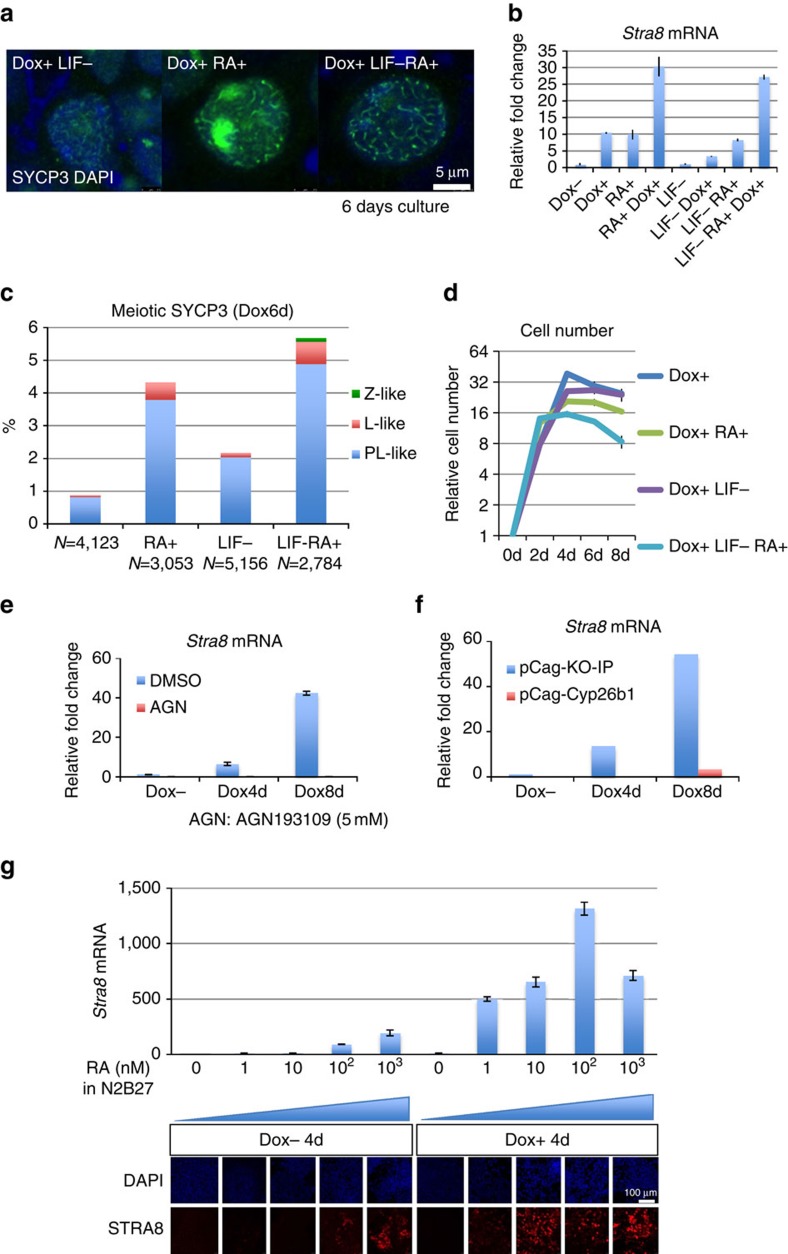
RA treatment potentiates meiosis-related changes in *Max* expression-ablated ESCs. (**a**) Representative images of meiosis-like SYCP3-staining patterns in Dox-treated *Max*-null ESCs cultured with or without RA in the presence or absence of LIF for 6 days. (**b**) Expression levels of the *Stra8* gene in Dox-treated *Max*-null ESCs cultured under the indicated conditions for 6 days. The *Stra8* gene expression level in Dox-untreated control *Max*-null ESCs cultured in the presence of LIF was arbitrarily set to one (*n*=3, means±s.d.). (**c**) Frequency of meiosis-like SYCP3-staining patterns in *Max*-null ESCs treated with Dox for 6 days or together with RA in the presence or absence of LIF. (**d**) Effect of RA on the recovery of viable Dox-treated *Max*-null ESCs cultured with or without LIF. The number of cells at day 0 was arbitrarily set to one (*n*=3, means±s.d.). (**e**) Quantification of *Stra8* mRNA levels in Dox-treated or -untreated *Max*-null ESCs cultured in the presence or absence of the RA receptor inhibitor AGN for the indicated days (*n*=3, means±s.d.). (**f**) Effect of the expression of *Cyp26b1* on *Stra8* expression levels in *Max*-null ESCs. (**g**) Dose-dependent effect of RA on *Stra8* expression levels in *Max*-null ESCs cultured in N2B27 with no RA as basal medium (*n*=3, means±s.d.).

Retinoid^7^,^8^,^9^,^10^ and LIF^36^ signalling are critical players in the onset of meiosis in germ cells. We thus decided to examine the effects of RA and LIF on *Max*-null ESCs. Similarly to vitamin C, RA treatment further enhanced the meiosis-related changes in *Max*-null ESCs (Fig. 6a–c). With respect to LIF, although its withdrawal from culture showed little to no effect on *Stra8* expression, it facilitated meiosis-related cytological changes in *Max*-null background (Fig. 6c). We also found that the number of viable cells was inversely correlated with the magnitude of meiosis-like changes (Fig. 6d), again implying that meiosis-like changes in ESCs are accompanied by apoptotic cell death. Our analyses with the RA receptor inhibitor AGN193109 (AGN; Fig. 6e and Supplementary Fig. 9a,b) and forced expression of *Cyp26b1* encoding an RA-degrading enzyme (Fig. 6f, Supplementary Fig. 9c,d) also confirmed the crucial involvement of RA, likely present in fetal bovine serum used, in meiotic changes in *Max* expression-ablated ESCs. To confirm this, we conducted experiments using serum- and RA-free N2B27 medium. Without exogenous supply of RA, induction of *Stra8* was barely detectable, irrespective of the presence or absence of *Max* expression in N2B27 medium. Interestingly, RA supplementation augmented *Stra8* expression levels dose-dependently even in the presence of *Max* expression, although much more profound induction was attained in the absence of *Max* expression. These results suggest that RA is essential for the induction of *Stra8* expression and that *Max* expression-ablated background sensitized ESCs to the RA-mediated change (Fig. 6g).

### Blockade of meiosis by MAX/MGA-containing atypical PRC1

**Figure 7:**
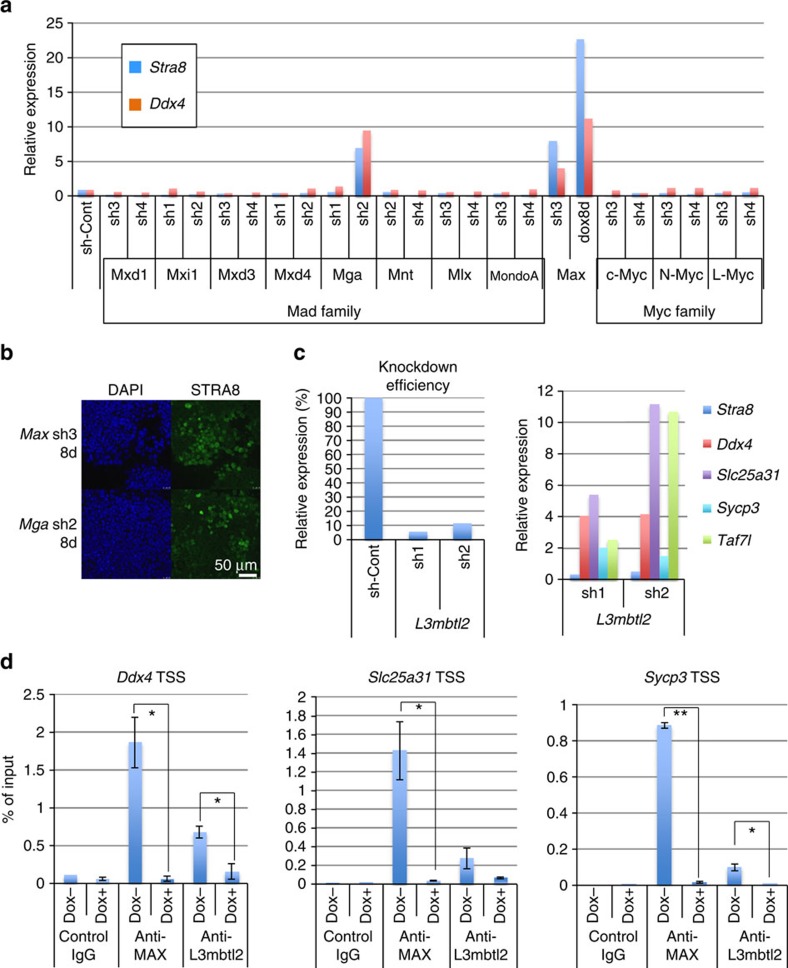
Implication of the MAX-containing PRC1.6 complex in the blockade of meiosis-related gene expression in ESCs. (**a**) Effect of knockdown of *Myc* superfamily genes on the expression of *Stra8* and *Ddx4* genes in ESCs. Expression levels of *Stra8* and *Ddx4* genes were quantitated in Dox-untreated *Max*-null ESCs subjected to lentivirus-mediated stable knockdown of the indicated genes. Expression levels of *Stra8* and *Ddx4* genes in cells subjected to knockdown with a scrambled sequence was arbitrarily set to one. Knockdown efficiency of each gene is shown in Supplementary Fig. 10a. (**b**) Immunocytochemical analyses of STRA8 in Dox-untreated *Max*-null ESCs in which expression of *Max* (upper) and *Mga* (lower) was subjected to lentivirus-mediated stable knockdown. (**c**) Effect of *L3mbtl2* knockdown on meiotic gene expression in ESCs. Lentivirus-mediated knockdown was conducted as described in **a.** Expression of *Stra8*, *Ddx4*, *Slc25a31*, *Sycp3* and *Tafl7* was determined by TaqMan-based quantitative PCR. (**d**) ChIP analyses of meiotic gene promoters with antibodies against MAX and L3MBLT2. Dox-untreated and -treated *Max*-null ESCs for 4 days were subjected to ChIP with either anti-MAX or L3MBLT2 antibodies. A ChIP reaction was also performed with control IgG. The genomic DNAs recovered by these ChIP reactions were individually subjected to quantitative PCR to quantify the relative amounts of *Ddx4*, *Slc25a31* and *Sycp3* gene promoters compared with those used as input samples (*n*=3, means±s.d.). Unpaired *t*-test was done to compare immunoprecipitation efficiency statistically between Dox-untreated and -treated *Max*-null ESCs (*P<0.05; **P<0.01).

To explore the molecular mechanisms underlying the meiosis-related changes associated with *Max* depletion in ESCs, we first searched for factors in the MYC superfamily, whose deficiency caused meiosis-related changes in ESCs, similar to MAX ablation. We found that lentivirus-mediated knockdown of *Mga* and *Max*, but not those of other MYC superfamily members, were coupled with induction of the expression of meiotic genes *Stra8*, *Taf7l* and *Slc25a31* (Fig. 7a and Supplementary Fig. 10). *Mga* knockdown-mediated induction of STRA8 expression was also confirmed by immunostaining (Fig. 7b). Because both MAX and MGA were recently demonstrated to be a part of an atypical PRC1 complex (PRC1.6)^14^,^15^, we next examined the consequence of forced reduction of the expression of the *L3mbtl2* gene that also encodes one of the components of PRC1.6. Knockdown of *L3mbtl2* expression also led to significant elevation in the expression levels of meiotic genes, although the expression levels of the *Stra8* gene were not affected (Fig. 7c). Next, to assess whether elevation of meiotic gene expression is the direct consequence of *Max* expression ablation or secondary in nature, we examined whether MAX protein binds to the meiosis-related genes. To this end, we conducted chromatin immunoprecipitation (ChIP) analyses with an anti-MAX antibody. Our data clearly demonstrated that MAX bound to *Ddx4*, *Slc25a31* and *Sycp3* genes in Dox-untreated ESCs (Fig. 7d). However, such signals were attenuated on *Max* expression ablation by Dox treatment. These results indicated that the MAX-containing complex directly bound to meiosis-related genes and repressed their expression in ESCs, and upregulation of these genes in *Max*-null ESCs reflects the liberation from such MAX-dependent repression. We also conducted ChIP analyses with an antibody against L3MBTL2. Although the immunoprecipitation efficiency was not as high with the antibody against L3MBTL2 compared with the anti-MAX antibody, our data indicated that L3MBTL2 directly bound to the examined meiotic genes. Furthermore, our data demonstrated that the binding signal was greatly diminished on *Max* expression ablation, implying that binding of L3MBTL2 on the meiotic genes is dependent on the presence of MAX (Fig. 7d). This finding further supported the notion that PRC1.6 complex is involved in the repression of meiotic genes in ESCs. Next, we examined the expression levels of genes encoding the *Myc* superfamily and those encoding either one of the components of the PRC1.6 complex after *Max* expression ablation in ESCs. We found that many of the genes, including *L3mbtl2*, showed slight decline in their expression levels following *Max* depletion in ESCs, while expression level of *Mga* was initially elevated (up to 4 days) but declined thereafter (Supplementary Fig. 11).

### Meiosis-like changes in ESCs with a MYC/MAX complex

**Figure 8:**
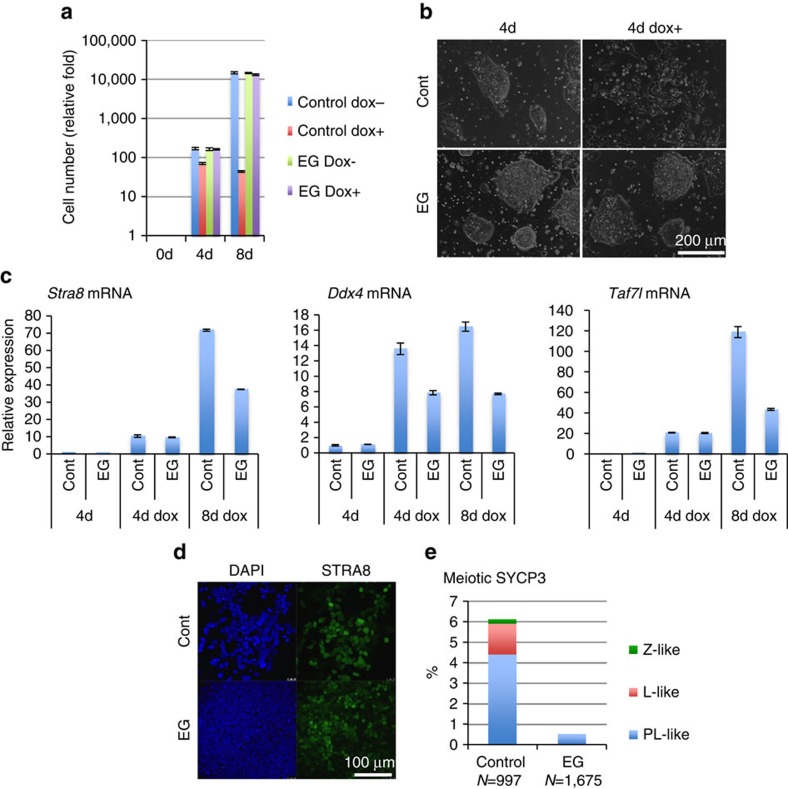
Meiosis-like changes in Dox-treated *Max*-null ESCs with rescued viability by mutant c-MYC/MAX. (**a**) *Max*-null ESCs with or without mutant c-MYC/MAX, whose expression was under the control of the constitutive β-actin promoter, were treated with Dox for the indicated days, and then viable cell numbers were counted. The number of cells at day 0 (0d) was arbitrarily set to one (*n*=3, means±s.d.). (**b**) Microscopic inspection of Dox-treated and -untreated *Max*-null ESCs for 4 days (4d) with or without mutant c-MYC/MAX. (**c**) Examination of the expression levels of meiotic genes in Dox-treated and untreated *Max*-null ESCs with or without mutant c-MYC/MAX. Expression levels of *Stra8*, *Ddx4* and *Tafl7l* were examined by TaqMan-based quantitative PCR (*n*=3, means±s.d.). (**d**) *Max*-null ESCs with or without mutant c-MYC/MAX were treated with Dox for 8 days (8d) and then subjected to immunostaining with antibodies against STRA8. (**e**) Frequency of the meiosis-like SYCP3-staining pattern in Dox-treated *Max*-null ESCs with or without mutant c-MYC/MAX for 8 days. Cont, control.

We have previously demonstrated that the detrimental phenotype of *Max*-null ESCs could be rescued by c-MYC and MAX mutants bearing modifications in their leucine zipper dimerization domains^16^,^36^. Because of these modifications, c-MYC and MAX mutants, designated as c-MYC-EG and MAX-EG, respectively, do not bind to other endogenous partners, but dimerize efficiently with each other to function as a c-MYC/MAX transcriptional complex. Therefore, *Max*-null ESCs bearing these mutants are able to maintain MYC activity but lack a functional PRC1.6 complex because the MAX-EG mutant does not interact with the endogenous MGA protein. We examined whether meiosis-like changes were evident in *Max*-null ESCs expressing c-MYC-EG and MAX-EG. Consistent with our previous report, *Max*-null ESCs bearing these mutants were able to maintain cell viability (Fig. 8a,b). However, our analyses revealed that the induction of expression of meiotic genes and production of STRA8 protein were also observed in these cells (Fig. 8c,d). Furthermore, meiosis-like cytological changes were detected as well (Fig. 8e). These meiotic-like changes were found, however, to a lesser degree when compared with control *Max*-null ESCs. These results suggest that a PRC1.6-independent MYC/MAX activity may also help guard meiosis entry in ESCs.

### *In vivo* meiosis is coupled with a decline in *Max* expression

**Figure 9:**
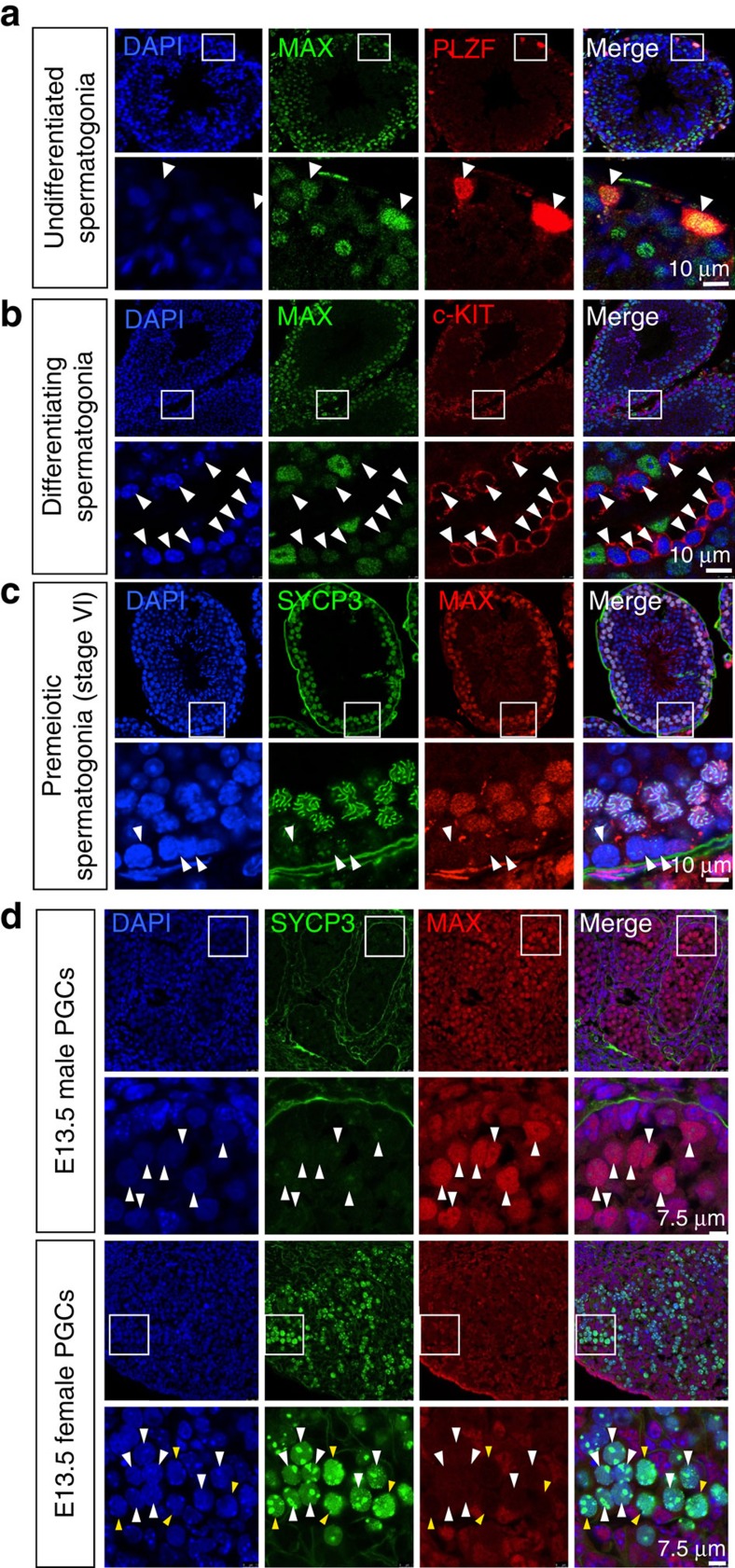
*Max* expression dynamics in normal gametogenesis. Frozen sections of testes from adult male mice were prepared and immunostained for MAX. The sections were also co-immunostained for PLZF (**a**), cKIT (**b**) or SYCP3 (**c**). Arrowheads in **a**,**b** and **c** indicate PLZF-positive undifferentiated spermatogonia that are strongly positive for MAX, cKIT-positive differentiating spermatogonia that are weak for MAX and cells undergoing meiotic cell division (presumable premeiotic type B spermatogonia) that are weak for both SYCP3 and MAX, respectively. (**d**) Decline of MAX expression levels in female, but not in male, PGCs of 13.5 dpc embryos. Male and female embryos (13.5 dpc) were recovered from a single pregnant female mouse, and then the genital ridges were recovered individually. After processing, sections were prepared and subjected to co-immunostaining with antibodies against MAX and SYCP3. Arrowheads shown in male genital ridges represent PGCs with strong and weak signals for MAX and SYCP3, respectively. Corresponding female PGCs with strong expression of SYCP3 are also indicated by arrowheads in which white and yellow indicate weak and almost no MAX expression, respectively.

Since meiosis-like cytological changes could be induced in *Max* expression-ablated ESCs, we decided to examine whether *Max* expression levels decline during normal meiotic cell division in testes. To this end we performed immunohistochemical analyses of testis sections. In addition to an antibody against MAX, antibodies against PLZF and cKIT, which mark spermatogonial stem/progenitor cells and differentiating spermatogonia, respectively, were used in the analyses to facilitate staging of cells undergoing meiosis^37^. We found that MAX expression, which is generally thought to be ubiquitous^38^, was extremely low in cKIT-positive differentiating spermatogonia and premeiotic type B spermatogonia at stage VI compared with PLZF-positive spermatogonial stem/progenitor cells (Fig. 9a–c). This low level of MAX expression in differentiating spermatogonia persisted up to the early pachytene stage but increased again during the pachytene stage (Supplementary Fig. 12). Whereas the meiotic cell division begins in testes after birth in males, meiosis commences in the genital ridges at the midgestation stage in females^39^. Therefore, we examined whether the meiotic processes in female mice are also accompanied by downregulation of MAX expression. Indeed, we found that MAX expression levels were extremely low in SYCP3-positive female PGCs at 13.5 dpc, which coincides with meiosis initiation, but were much higher in E13.5 male PGCs (Fig. 9d). These results reveal that, although occurs at different timing, meiosis in both sexes is coupled with downregulation of MAX expression. To assess whether there is also a difference in *Max* mRNA levels between male and female PGCs, we inspected DNA microarray data of PGCs deposited by Kagiwada *et al.*^40^ We found that *Max* expression levels in female PGCs was lower compared with those of male PGCs, especially at the initiation of meiosis (13.5 dpc; Supplementary Fig. 13a), implicating that the lower level of MAX protein in female PGCs revealed by immunostaining was at least in part due to lower levels of *Max* mRNA in female PGCs compared with that in male PGCs. However, our data do not exclude the possibility that MAX protein levels were also controlled post-translationally.

We also used these DNA microarray data to study the expression dynamics of genes whose expression levels were elevated in *Max*-null ESCs. Both germline-specific genes and those categorized into both germline and two-cell signature genes shown in Supplementary Fig. 1 also had strong elevations in their expression levels during midgestation (E10.5–13.5; Supplementary Fig. 13b). Interestingly, most meiotic genes showed significant elevations in their expression levels not only in female PGCs but also in male PGCs during gestation, although the magnitude of the elevation was not as prominent as that observed in female PGCs. More remarkably, most of the genes that were significantly or modestly activated in *Max*-null ESCs were also, respectively, activated in similar manners during PGCs development, providing additional evidence that our *Max* expression ablation system in ESCs represents a *bona fide* model of meiosis. However, genes categorized exclusively as two-cell signature genes did not show any tendency of activation, but rather decreased their expression levels during PGCs development. These results further suggest that activation of these genes in *Max* expression-ablated ESCs is not related to meiosis-like changes (Supplementary Fig. 13b).

### Meiotic induction in GSCs by *Max* knockdown

**Figure 10:**
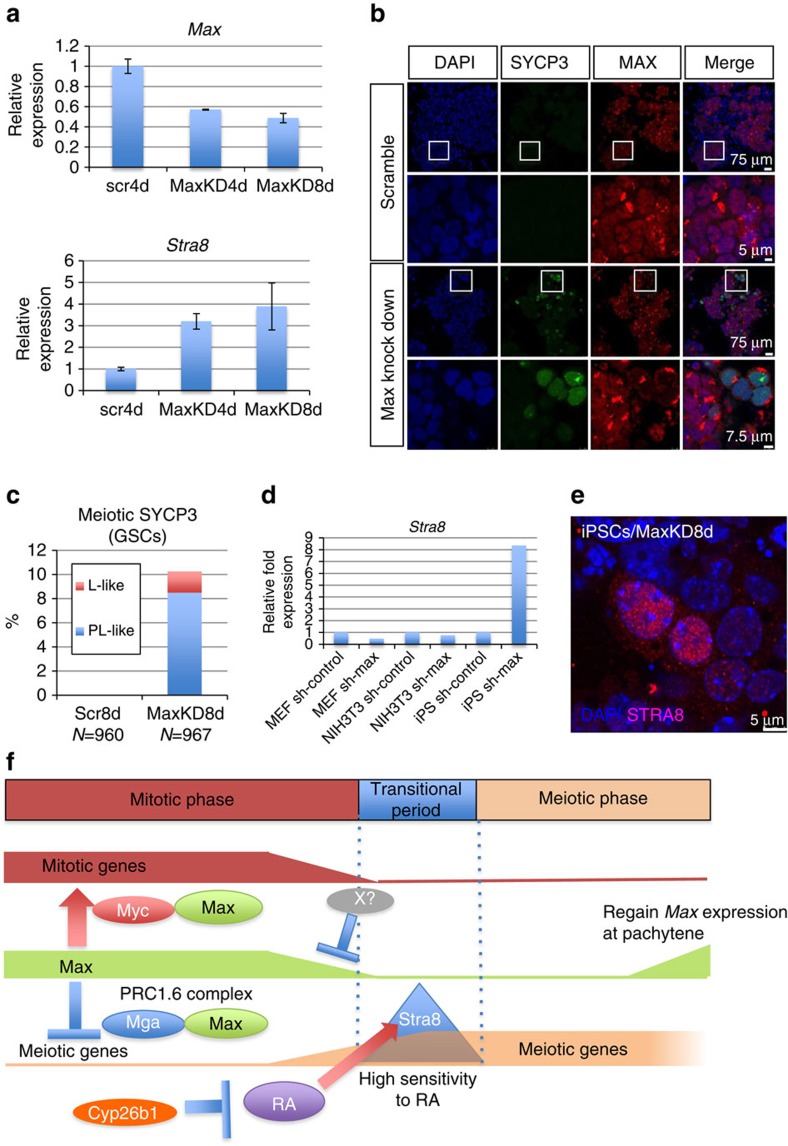
Induction of meiosis-like changes in GSCs by knockdown of *Max* expression. (**a**) Effect of *Max* knockdown in GSCs on expression of the *Stra8* gene. Lentivirus-mediated knockdown was conducted as described in Fig. 7. Upper panel shows the knockdown efficiency of *Max* expression (*n*=3, means±s.d.). (**b**) Effect of *Max* knockdown in GSCs on induction of the meiosis-like SYCP3-staining pattern. GSCs were subjected to knockdown with scrambled or specific sequences for *Max* expression and then applied to co-immunostaining analyses with antibodies against MAX and SYCP3. (**c**) Frequency of the meiosis-like SYCP3-staining pattern in GSCs subjected to lentivirus-mediated knockdown of *Max* expression. (**d**) Effect of *Max* knockdown in MEFs, NIH3T3 cells and iPSCs on the induction of *Stra8* expression. The levels of *Stra8* expression were measured by quantitative PCR. (**e**) Immunocytochemical analyses of iPSCs subjected to lentivirus-mediated knockdown of *Max* expression with an antibody against STRA8. (**f**) Schematic representation of possible inverse regulation of the expression of meiotic and mitotic genes through the control of *Max* expression for efficient meiosis. MAX is known to play important roles in preserving the stem cell properties of ESCs and GSCs by functioning as an obligated partner for MYC transcription factors. Our present study demonstrates that MAX also contributes to the maintenance of stemness of these cells by blocking expression of meiotic genes. Artificial and physiological declines in *Max* expression levels in ESCs and GSCs, respectively, enhance and reduce expression of meiotic and mitotic genes, respectively, through liberation from the MAX/MGA-dependent repression mechanism and the loss of MYC/MAX complex-dependent transcriptional activation. The former potentiates meiotic progression, whereas the latter results in disruption of the stem/progenitor properties and, therefore, facilitates cellular differentiation. Our data suggest that the MAX-containing PRC1.6 complex is the molecule responsible for the MAX-mediated repression of meiotic genes. However, it is unknown at present what lowers *Max* expression levels at the onset of meiosis in GSCs.

We next examined the consequence of forced reduction of *Max* expression in GSCs. We not only found that *Stra8* expression level was elevated (Fig. 10a) but also detected cytological changes reminiscent of meiosis in GSCs on *Max* knockdown (Fig. 10b,c). Of note is that these cytological changes were only observed in cells with reduced *Max* expression. Because knockdown of *Max* expression was insufficient, but *Max* expression should be completely ablated in ESCs to induce meiosis-related cytological changes, our data with GSCs suggested that GSCs are more competent for meiosis-like cytological changes than ESCs. Next, we conducted knockdown of *Max* expression in mouse embryonic fibroblasts, NIH3T3 cells and induced pluripotent stem cells (iPSCs). Although lentivirus-mediated knockdown of *Max* expression enhanced *Stra8* gene expression levels in iPSCs, no such induction was evident in mouse embryonic fibroblasts or NIH3T3 cells (Fig. 10d). Induction of STRA8 in iPSCs was confirmed by immunostaining (Fig. 10e). Therefore, we conclude that meiosis-related changes due to *Max* expression deficiency are specific to germ and pluripotent cells.

## Discussion

In this study we have uncovered an intriguing meiosis-like programme in ESCs triggered by loss of function of *Max*. Our results show that the loss of *Max* expression in ESCs not only activates germ cell-related genes but also leads to cytological changes resembling leptotene and zygotene stages of meiosis.

Our functional screening of the *Myc* superfamily led to the identification of MGA, whose absence also triggered the meiosis-like changes. Our subsequent analyses revealed that a MAX-containing atypical PRC1 complex (PRC1.6) is responsible for the molecular blockade of meiotic onset. There are six distinct PRC1 complexes with variations in their components, but only in PRC1.6 (ref. 14) are both MAX and MGA present. A unique characteristic of PRC1.6 is that this variant PRC1 does not co-localize with PRC2 at their target sites^15^. Therefore, PRC1.6 must bind to DNA stably without PRC2, and possible molecules responsible for such stable DNA binding include MAX and its association factor MGA. In this context, it is of interest to mention that the MAX-containing PRC1 complex has been shown to be linked to the E box sequence that serves as a binding site for MAX and MGA^15^.

We provide evidence of the existence of a transient decline in *Max* expression during physiological gametogenesis in both male and female germ cells. This lead us to assume that the same or at least a similar process of MAX-dependent blockade of meiosis is present in GSCs. GSCs should be liberated from such blockades by a physiological decline in *Max* expression to proceed with meiosis during their differentiation. This assumption was further corroborated by data demonstrating that knockdown of *Max* expression in GSCs also led to meiosis-like cytological changes. It is noteworthy that MYC family proteins, including c-MYC and N-MYC, play crucial roles in preservation of the self-renewal properties of both ESCs^41^ and GSCs^42^ in which MAX functions as an indispensable partner. Thus, as depicted in Fig. 10f, loss of MAX may promote meiosis not only by activating the expression of meiotic genes but also by facilitating cellular differentiation through impairment of the self-renewal of ESCs and GSCs because of the loss of MYC functions. The differences in efficiencies in the induction of meiosis-like changes between *Max*-null ESCs bearing c-MYC, MAX mutants and control *Max*-null ESCs may be explained by the preservation of MYC functions in the former, but not in the latter cells. It is noteworthy that Yokobayashi *et al.*^43^ have demonstrated that the PRC1 complex acts negatively against RA signalling through maintenance of the transcriptionally repressed state of the *Stra8* gene. Similarly, our data demonstrated that *Max* expression ablation in ESCs, which is coupled to disruption of the PRC1.6 complex, sensitized RA-mediated induction of *Stra8* gene expression. This finding further strengthens the notion that the meiotic onset in ESCs faithfully recapitulates the *in vivo* meiotic onset. Observations made by Yokobayashi *et al.*^43^ on the loss of function of PRC1 in germ cells may not reflect impairment of the general functions of PRC1, but might be due to disruption of the MAX-containing atypical PRC1 complex.

*Max* expression ablation appears to induce meiotic onset in ESCs in a physiologically relevant manner. However, such meiosis-like cells are arrested at rather early stages and do not proceed beyond the crux of the pachytene-like stage. To overcome this problem, development of improved culture conditions suitable for meiosis and/or the forced expression of inefficiently induced meiotic genes such as *Rec8* and *Sycp1* will be needed. Although we are far from a complete understanding of the mechanisms involved in generating haploid cells, the results and methodologies here described may open up new avenues towards achieving this goal.

## Methods

### Animal ethics

This study was carried out under strict accordance with the international and institutional guidelines. The protocol was approved by the institutional review boards on the Ethics of Animal Experiments of the Saitama Medical University, with the permission number of 1518.

### Mouse ESC and GSC culture

*Max*-null ESCs with Max cDNA under the control of the tetracycline-off system were cultured in conventional ESC culture medium with LIF and serum, or the 2i condition with inhibitors of MEK (PD0325901) and GSK3β (CHIR99021) with the concentrations of 1 and 3 μM, respectively. N2B27 not containing vitamin A was used as basal medium for the experiments shown in Fig. 6g. The following reagents were used at the indicated concentration unless stated otherwise: 1 μg ml^−1^ Dox (631311, Clontech); 100 nM VX680 (A10981-25, ADG); 20 μM Z-VAD-FMK (627610, Calbiochem); 5 μM AGN193109 (sc210768, Santa Cruz Biotechnology); 1 μM all-trans retinoic acid (R2625, Sigma); and 50 μg ml^−1^ vitamin C (A4544, SIGMA). Mouse GSCs were cultured with Stem Pro-34 SFM medium (10639-011, Invitrogen) with the ingredients of 25 μg ml^−1^ insulin (1882, Sigma), 100 μg ml^−1^ transferrin (Sigma, T1147), 60 μM putrescine (P7505, Sigma), 30 nM sodium selenite (214485, Sigma), 6 mg ml^−1^ D-(+)-glucose (G7021, Sigma), 30 μg ml^−1^ pyruvic acid (107360, Sigma), 1 μg ml^−1^ DL-lactic acid (4263, Sigma), 5 mg ml^−1^ bovine serum albumin (15561-020, Invitrogen), 2 mM L-glutamine, 50 nM 2-mercaptoethanol, MEM vitamin solution (11120-052, Invitrogen), 0.1 mM nonessential amino acid solution (11140-050, Invitrogen), 30 ng ml^−1^ β-estradiol (E2758, Sigma), 60 ng ml^−1^ progesterone (P8783, Sigma), 10 μg ml^−1^ D-biotin (B4501, Sigma), 100 mM ascorbic acid (A4544, Sigma), 20 ng ml^−1^ mouse epidermal growth factor (2028-EG, R&D systems), 10 ng ml^−1^ human basic fibroblast growth factor (13256-029, Becton Dickinson), 1,000 U ml^−1^ mouse leukaemia inhibitory factor (02740, Stem Cell Technologies), 10 ng ml^−1^ rat glial cell line-derived neurotrophic factor (512-GF, R&D systems) and 1% fetal bovine serum.

### Plasmid constructions

Construction of expression vectors for c-MYC and MAX mutants, designated as c-MYC-MG and MAX-EG, respectively, which do not bind to their endogenous partners, but bind efficiently with each other, was described previously^16^,^36^. CYP26B1 expression vector was constructed by PCR amplification using cDNA from mouse ESCs and subcloned into pCAG-IP vector bearing puromycin resistant gene.

### Quantitative reverse transcription (RT)–PCR analysis

Quantitative RT–PCR (qRT–PCR) was performed using the StepOnePlusTM Real-Time PCR System (Applied Biosystems), with cDNAs generated by reverse transcription. TaqMan-based reactions (Invitrogen) quantified the expression levels of *Stra8* (Mm00486473_m1), *Slc25a31* (Mm00617754_m1), *Sycp3* (Mm00488519_m1), *Taf7l* (Mm00459354_m1), *Ddx4* (Mm00802445_m1), *c-Myc* (Mm00487803_m1), *N-Myc* (Mm00476449_m1), *L-Myc* (Mm03053598_s1) and *Max* (Mm00484802_g1), while qRT–PCR for other genes, including quantification of other *Myc* superfamily genes such as *Mxd1* (Supplementary Fig. 10), was done by SYBR Green-based method with the following oligonucleotides.

*L3mbtl2*: 5′-TACCTCATGAAGCGGTTGGT-3′ and 5′-CAACCCACTGGGTGGATC-3′

*Mxd1*: 5′-AGATGCCTTCAAACGGAGGAA-3′ and 5′-CAAGCTCAGAGTGGTGTGTCG-3′

*Mxi1*: 5′-AACATGGCTACGCCTCATCG-3′ and 5′-CGGTTCTTTTCCAACTCATTGTG-3′

*Mxd*3: 5′-GAGGCAGAGCACGGTTATG-3′ and 5′-TGTAGTGTATCGGGTACAGTCAA-3′

*Mxd4*: 5′-ATGGAGCTGAACTCTCTGCTG-3′and 5′-GTGAAGACCTGTTGTTCGGGG-3′

*Mga*: 5′-GAGGAGCACCTACCTACCTTCTTTGT-3′ and 5′-ACGGGCATCTCGATTAGTAACT-3′

*Mnt*: 5′-TCCTGTAGTGACCAATTCCCC-3′ and 5′-TGGCTCCTTAATGCTGAGTCC-3′

*Mlx*: 5′-GAGGACAGTGATTATCAGCAGGA-3′ and 5′-CATCCCTTCTCTTCTGTTCAGC-3′

*MondoA*: 5′-GACTCGGACACAGACGAGC-3′and 5′-GGAGAGGACACCATGAAGTGG-3′

### Immunostaining

For immunocytochemistry, cells were plated on 0.1% gelatin-coated Cell Disks (MS-92132, SUMITOMO BAKELITE Co., Tokyo, Japan) and cultured for an appropriate period. Then the cells were fixed with 4% paraformaldehyde for 20 min at room temperature. After extensive washes with phosphate-buffered saline (PBS), the cells were permeabilized and blocked in blocking buffer (PBS containing 0.3% Triton X-100, and 5% fetal bovine serum) for 60 min at room temperature. Subsequently, the cells were washed with PBS and then incubated with primary antibodies in blocking buffer at 4 °C overnight. After three washes with PBS, the cells were incubated with the appropriate Alexa Fluor dye-conjugated secondary antibodies (Invitrogen) and 3 μg ml^−1^ 4′,6-diamidino-2-phenylindole (DAPI) in blocking buffer at 37 °C for 1 h. The cells were washed and observed under a confocal laser scanning microscope (TCS SP8, Leica Microsystems) and analysed with ImageJ computer software. For immunohistochemistry, male testes and the genital ridges of male and female siblings were isolated from 8-week-old male mice and 13.5 dpc embryos, respectively. After fixing with 4% paraformaldehyde, the tissues were embedded in optimal cutting temperature (OCT) compound and frozen. Sections prepared from the frozen tissues were mounted on glass slides and subjected to immunostaining procedures as described above. The primary antibodies used were as follows: anti-SYCP1 (1:400, ab15090), anti-SYCP3 (1:400, ab97672), anti-STRA8 (1:400, ab49602), anti-REC8 (1:100, ab192241) and anti-Aurora B (1:1,000, ab2254) from Abcam; anti-MAX (1:100, sc-197), anti-L3mbtl2 (1:100, sc-134879) and anti-PLZF (1:100, sc28319) from Santa Cruz Biotechnology; 5-methylcytosine (1:100, 61255) and 5-hydroxymethylcytosine (1:100, 39791) from Active Motif; anti-γH2AX (1:1,000, #2577, Cell Signaling Technology); anti-cKIT (1:100, 553352) from BD Pharmingen; and Anti-Aurora A/B/C (1:300, BS-2445R) from Bioss Antibodies.

### Estimation of meiotic stages from SYCP3-staininig length

Germ cells in preleptotene, leptotene, zygotene, pachytene and diplotene stages were identified the ordinary way, with testis sections immunostained and counterstained with anti-SYCP3 antibody and DAPI, respectively. Subsequently, actual lengths of SYCP3-staininig regions were measured with respect to five representative chromosomes of each cell. Some cells with large dots, the width of which was more than 1.5 μm, often found in cells at preleptotene and leptotene stages, were eliminated from the analyses. Exploration of the relationship between averages of these measurements and meiotic stages led to the following criteria: preleptotene-like, <1.2 μm; leptotene-like, 1.2∼3.0 μm; zygotene-like, 3.0∼7.5 μm; and pachytene or diplotene-like, >7.5 μm. This method did not allow us to distinguish between pachytene and diplotene stages. These criteria were used to assign the stages of meiosis-like cells from *Max*-null ESCs. For details, see Supplementary Fig. 3.

### Gene disruption

The CRISPR/Cas9 system was used to conduct homozygous knockout of *Stra8* and *Blimp1* genes. The following oligonucleotides were used to edit exon 2 and exon 3 of *Stra8* and *Blimp1* genes, respectively.

*Stra8*: 5′-CACCGATAATGGCCACCCCTGGAGA-3′ and 5′-AAACTCTCCAGGGGTGGCCATTATC-3′.

*Blimp1*: 5′-CACCGATCATGAAAATGGACATGG-3′ and 5′-AAACCCATGTCCATTTTCATGATC-3′.

Sequences from the original genomic DNA are underlined. The oligonucleotides were annealed and subcloned into the Bbs1 site of the pX330-U6-Chimeric_BB_CBh-hSpCas9 vector^44^. The vector was then co-transfected with pCAG-IP into *Max-*null ESCs using lipofectamine 2000 (Invitrogen, Life Technology) and then subjected to puromycin selection. The resulting ESC colonies were individually cloned and used to prepare cell stocks and genomic DNA. Subsequently, PCRs were conducted to identify clones with homozygous knockout by direct DNA sequencing of the PCR products. Details of mutations in *Stra8* and *Blimp1* genes are shown in Fig. 4a and Supplementary Fig. 8, respectively.

### Lentivirus-mediated knockdown

Lentivirus-mediated knockdown of the expression of *Myc* superfamily genes and the *L3mbtl2* gene was conducted as described previously^45^. Sequences used for knockdown are shown in Supplementary Table 1.

### ChIP-PCR analyses

ChIP analyses were done with Dox-untreated and -treated *Max*-null ESCs for 4 days using antibodies against MAX or L3MBTL2. Primers used for amplifying promoter regions of *Ddx4*, *Slc25a31* and *Sycp3* genes, which were described by Maeda *et al.*^11^, Okashita *et al.*^46^ and Yamaguchi *et al.*^28^, respectively, are as follows:

*Ddx4*: 5′-ACTCACCTCTCCGCTCCAG-3′ and 5′-GTCGCCTGATGCTATTTGTTGT-3′.

*Slc25a31*: 5′-AAAGCTGCTGTGCACTGATTG-3′ and 5′-CTGAGGATGCTGGGAGAACAG-3′.

*Sycp3*: 5′-GGGGCCGGACTGTATTTACT-3′ and 5′-CTCCCCCATCTCCTTACCTC-3′.

### DNA microarray and GO analyses

Biotin-labelled cRNA was synthesized and then hybridized to Affymetrix GeneChip Mouse Genome 430 2.0 arrays (Affymetrix) according to the manufacturer's instructions. Microarray expression data were background subtracted and normalized by the robust multi-array analysis method using statistical software R. GO analyses were conducted using DAVID web tools (http://david.abcc.ncifcrf.gov). To eliminate redundancy, the selected GO terms were subjected to further analyses using AmiGO1 (http://amigo1.geneontology.org/cgi-bin/amigo/go.cgi) and REVIGO (http://revigo.irb.hr) web sites.

### DNA blot and bisulfite sequencing analyses

DNA blot analyses of global changes in 5mC and 5hmC due to *Max* expression ablation in ESCs were performed using genomic DNAs from Dox-untreated and -treated *Max*-null ESCs for 8 days as described by Blaschke *et al.*^29^. Bisulfite sequencing analyses of *Snrpn*, *Igf2r* and *H19* genes were performed with the same set of genomic DNAs using the following oligonucleotides, which are described by Lucifero *et al.*^47^

*Snrpn* first PCR: 5′-TATGTAATATGATATAGTTTAGAAATTAG-3′ and 5′-AATAAACCCAAATCTAAAATATTTTAATC-3′

*Snrpn* second PCR: 5′-AATTTGTGTGATGTTTGTAATTATTTGG-3′ and 5′-ATAAAATACACTTTCACTACTAAAATCC-3′

*Igf2r* first PCR: 5′-TTAGTGGGGTATAAAAGGTTAATGAG-3′ and 5′-AAATATCCTAAAAATACAAACTACAC-3′

*Igf2r* second PCR: 5′-GTGTGGTATTTTTATGTATAGTTAGG-3′ and 5′-AAATATCCTAAAAATACAAACTACAC-3′

*H19* first PCR: 5′-GAGTATTTAGGAGGTATAAGAATT-3′ and 5′-ATCAAAAACTAACATAAACCCCT-3′

*H19* second PCR: 5′-GTAAGGAGATTATGTTTATTTTTGG-3′ and 5′-CCTCATTAATCCCATAACTAT-3′

Uncropped versions of western blots shown in Fig. 4c and Supplementary Fig. 5a are shown in Supplementary Fig. 14a,b, respectively.

## Additional information

**Accession code:** DNA microarray data used in Fig. 5a have been deposited in the NCBI Gene Expression Omnibus under accession number GSE65700.

**How to cite this article:** Suzuki, A. *et al.* Loss of MAX results in meiotic entry in mouse embryonic and germline stem cells. *Nat. Commun.* 7:11056 doi: 10.1038/ncomms11056 (2016).

## Supplementary Material

Supplementary Information Supplementary Figures 1-14 and Supplementary Table 1.
