# Supplementary material for: Loss of MAX results in meiotic entry in mouse embryonic and germline stem cells
Source: Nat Commun. 2016 Mar 30;7:11056. doi: 10.1038/ncomms11056 (PMC4820925; doi:10.1038/ncomms11056)
Supplement: Supplementary Information — Supplementary Figures 1-14 and Supplementary Table 1. [file ncomms11056-s1.pdf]

Supplementary Figure Legends

Max cKO ESCs (Dox+)

Relative expression of Max null ESCs (Dox- =1)

	Gene Name	Dox1d	Dox2d	Dox3d	Dox4d	Dox6d	Dox8d
Meiosis	*Stra8*	2.96	1.67	7.72	29.15	38.90	45.42
	Dazl	5.00	1.71	21.09	49.16	43.23	41.21
	Ddx4	5.26	1.69	13.40	17.61	13.42	8.00
	Slc25a31	6.20	0.94	15.45	25.60	35.83	26.02
	Stk31	12.49	3.08	24.16	47.42	16.22	8.69
	Taf7l	4.71	1.13	21.81	32.48	42.20	37.63
	Tdrd9	0.97	0.72	5.88	16.17	20.17	7.82
	Sycp2	1.75	1.84	9.92	27.97	35.03	24.51
Meiosis & 2C*	*Sycp1*	3.20	0.92	8.57	19.64	18.38	20.97
	Sycp3	8.21	4.44	14.00	18.67	4.03	2.93
	Hormad1	13.81	3.21	31.74	56.20	12.77	8.16
	Mov10l1	1.84	0.80	7.54	12.79	23.52	19.64
	Tex101	1.02	0.71	11.89	16.40	23.52	36.54
	Tex11	6.68	3.86	25.06	32.02	9.35	6.38
	Tex15	3.91	3.81	12.08	37.09	33.16	21.74
2C	*Zscan4*	1.40	1.30	11.80	18.40	32.20	22.20
	Dub1	0.77	0.50	6.91	15.56	44.88	40.32
	Eif1a	1.84	2.37	15.01	25.20	29.53	27.38
	Gm4340	1.14	1.07	7.94	10.62	21.39	17.82
	Gm8300	2.24	2.54	25.96	48.79	41.80	36.99
	Tcstv3	1.07	0.73	8.62	14.77	24.81	23.09
	Zfp352	0.85	0.72	8.33	17.25	46.57	30.98

Relative expression of Max null ESCs (Dox- =1)

	Gene Name	Dox1d	Dox2d	Dox3d	Dox4d	Dox6d	Dox8d
PGC specification	*Blimp1*	1.46	1.99	3.03	3.09	1.56	0.71
	Dppa3	0.71	1.02	1.63	1.61	2.37	1.16
	Tcfap2c	0.99	1.60	0.82	1.40	2.87	1.42
Cohesin	*Smc1a*	1.73	2.17	1.88	2.37	0.98	0.68
	Smc1b	1.14	1.56	2.66	5.48	7.27	4.13
	Rad21	1.18	1.23	1.22	1.57	1.08	0.99
	Rec8	0.75	0.72	0.69	1.03	2.55	4.80
	Stag1	1.36	2.17	2.39	2.54	2.07	1.41
	Stag2	1.87	2.48	1.95	2.18	0.72	0.59
	Stag3	2.95	1.46	3.09	7.80	5.07	4.49
Recombination	*Spo11*	0.78	1.09	0.93	1.06	1.90	3.40
	Prdm9	0.98	0.70	1.29	1.19	1.62	1.83
	Rad51	0.85	0.90	0.92	0.79	0.81	0.95
	Mlh1	0.71	0.89	0.91	0.98	0.72	0.77
	Dmc1	1.48	1.76	1.75	2.59	2.09	1.41

Meiosis specific   Mitosis specific

0.02   50

Supplementary Figure 1 DNA microarray data of representative genes for germ cells and/or two-cell embryos. PGC specification genes such as *Blimp1* are shown separately from other germ cell marker genes. Expression data of genes encoding mitotic and meiotic cell division-specific cohesion components and genes for regulators of DNA recombination were also extracted from the DNA microarray data (GSE27881).

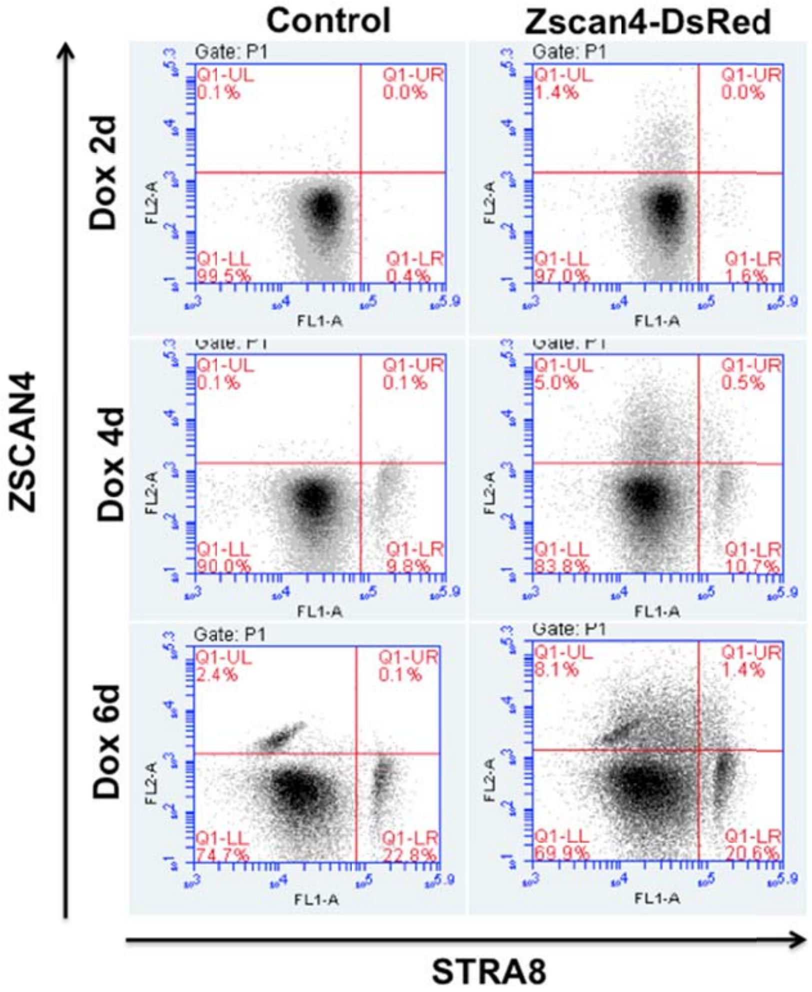

Supplementary Figure 2 Flow cytometric analyses to explore the relationship between activation of two-cell signature genes and meiosis-like changes in *Max*-null ESCs. *Max*-null ESCs with or without a stably integrated DsRed reporter gene, whose expression faithfully recapitulates endogenous *Zscan4* gene expression, were treated with Dox for the indicated days and then immunostained with an anti-STRA8 antibody and Alexa 488-labelled secondary antibody. Subsequently, these cells were subjected to flow cytometric analyses. Although it is unknown which type of cells among Dox-treated *Max*-null ESCs bearing no DsRed reporter gene were judged as DsRed positive, such cells are considered to be included among *Max*-null ESCs with the reporter. Therefore, the percentage of the latter cells was subtracted from that of the former cells to estimate the actual frequency of DsRed-positive cells. Flow cytometric analyses were done as described previously[46].

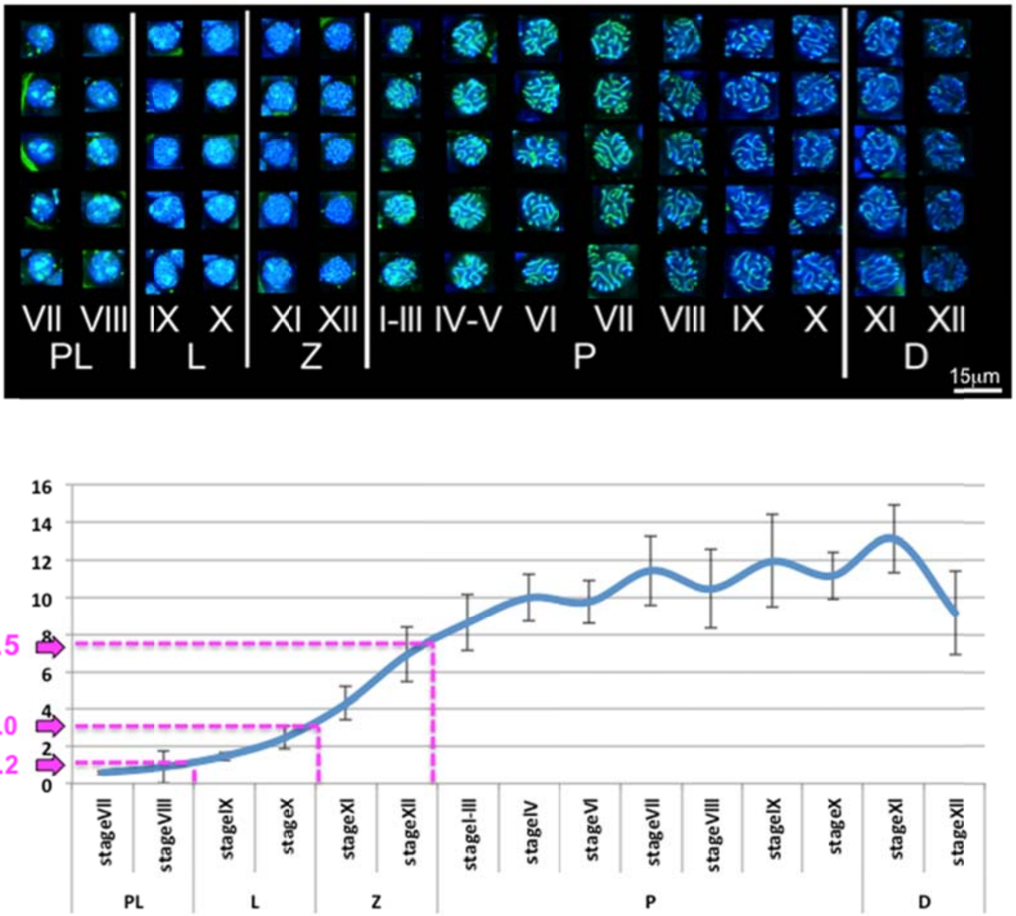

Supplementary Figure 3 Institution of criteria to distinguish the stages of meiosis by SYCP3-staining patterns. Testis sections were prepared from adult male mice, immunostained for SYCP3, and then counterstained with DAPI. The stage (I–XII) of the seminiferous epithelium cycle in each section was determined by the SYCP3-staining pattern and nuclear shape of spermatids. Subsequently, meiotic cells in preleptotene, leptotene, zygotene, pachytene and diplotene stages were identified based on the SYCP3-staining pattern and the location of each cell in each epithelial cell layer. Then, five representative chromosomes of each cell were used to measure lengths of SYCP3-staining regions and averages of these measurements were calculated with respect to the cells shown at the top and shown as means±s.d. Based on the data obtained from these analyses, three boundaries (1.2, 3.0 and 7.5 μm) were set for the average length of SYCP3 staining to distinguish among meiotic cells at preleptotene, leptotene, zygotene and pachytene stages, and used to assign the stages of meiosis-like cells derived from *Max*-expression ablated ESCs. Pachytene and diplotene stages were indistinguishable using these criteria.

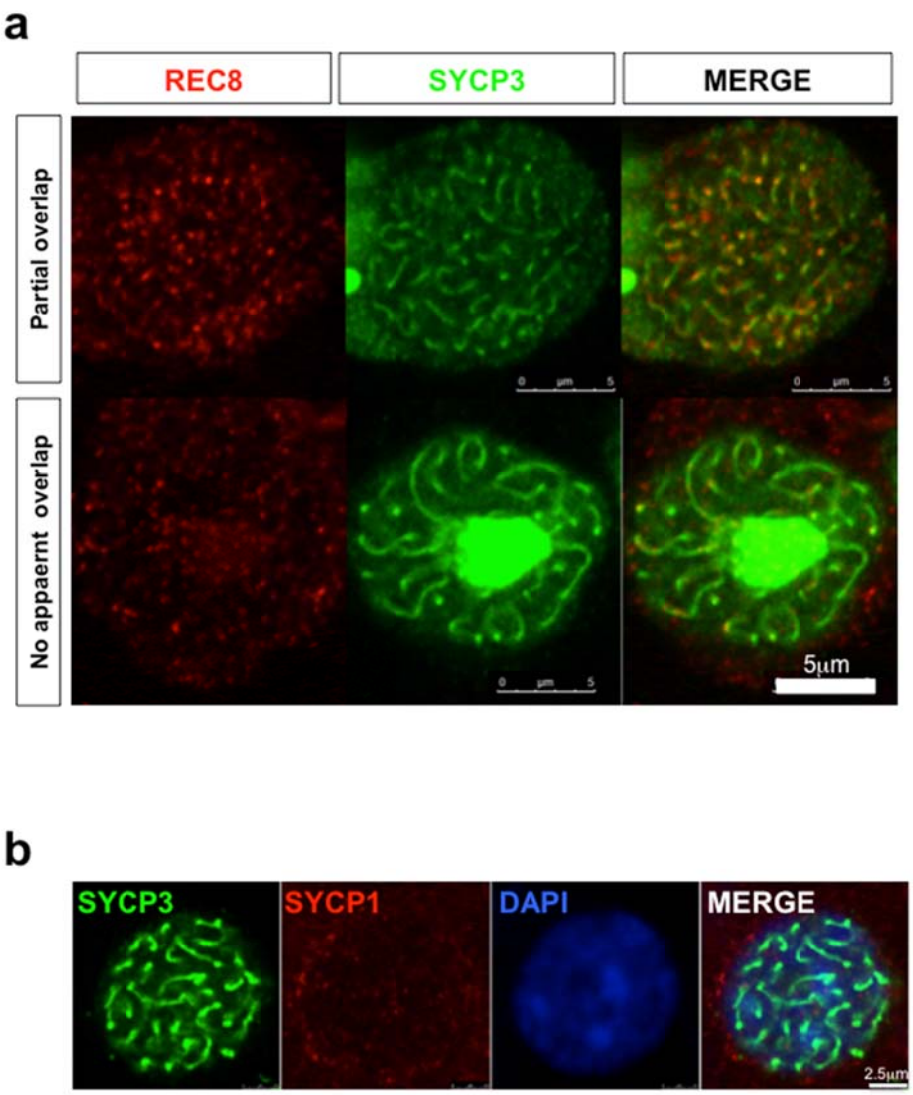

Supplementary Figure 4 Co-immunostaining analyses of Dox-treated *Max*-null ESCs. (**a**) Dox-treated *Max*-null ESCs were co-immunostained for SYCP3 and REC8 as described in Fig. 2. Upper and lower panels are representative images of partial and almost no overlaps between REC8 and SYCP3 staining, respectively. (**b**) Dox-treated *Max*-null ESCs were co-immunostained for SYCP3 and SYCP1 as described in **a**.

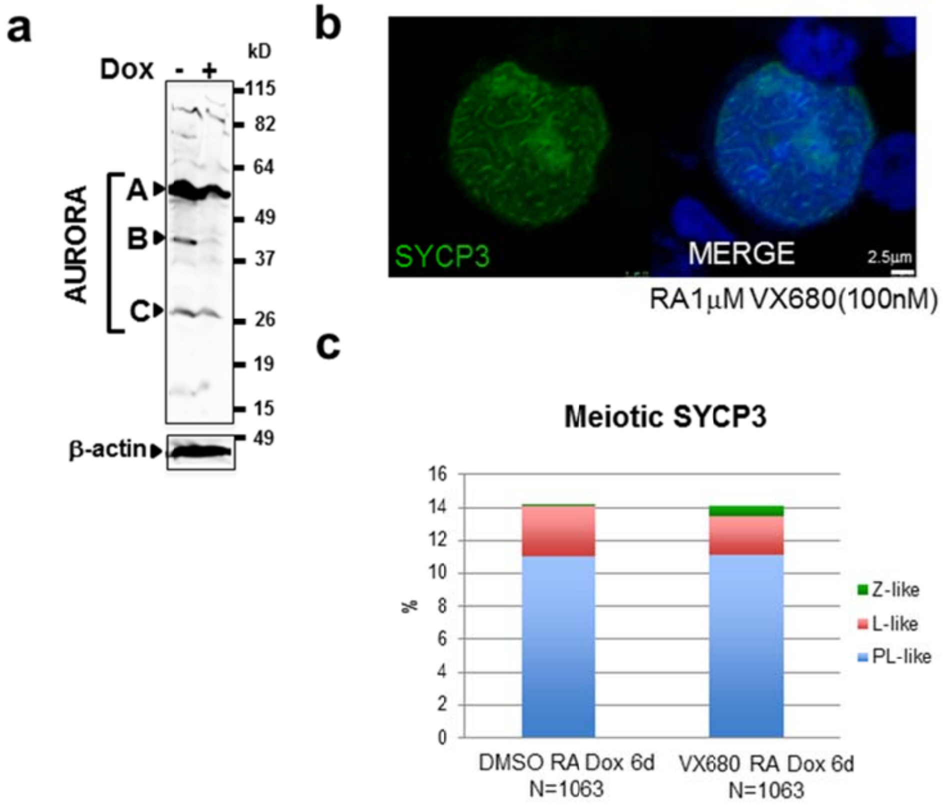

Supplementary Figure 5 Examination of the possible involvement of aurora kinases in the meiosis-like changes of *Max* expression-ablated ESCs. (**a**) Examination of the amounts of aurora kinases by western blot analyses. Whole cell extracts were prepared from *Max*-null ESCs untreated or treated with Dox for 8 days. The extracts were subjected to western blot analyses using an antibody that reacts with all three types of aurora kinases (A, B and C) after SDS-polyacrylamide electrophoresis and transfer to a polyvinylidene fluoride membrane. kD, kilodalton. (**b**) Effect of the pan-aurora kinase inhibitor VX680 on the meiosis-like changes in Dox-treated *Max*-null ESCs. *Max*-null ESCs were treated with Dox either in the presence or absence of VX680 (100 nM) for 6 days and then subjected to immunostaining with an antibody against SYCP3. (**c**) Frequency of meiosis-like SYCP3-staining patterns in *Max*-null ESCs treated only with Dox or together with VX680.

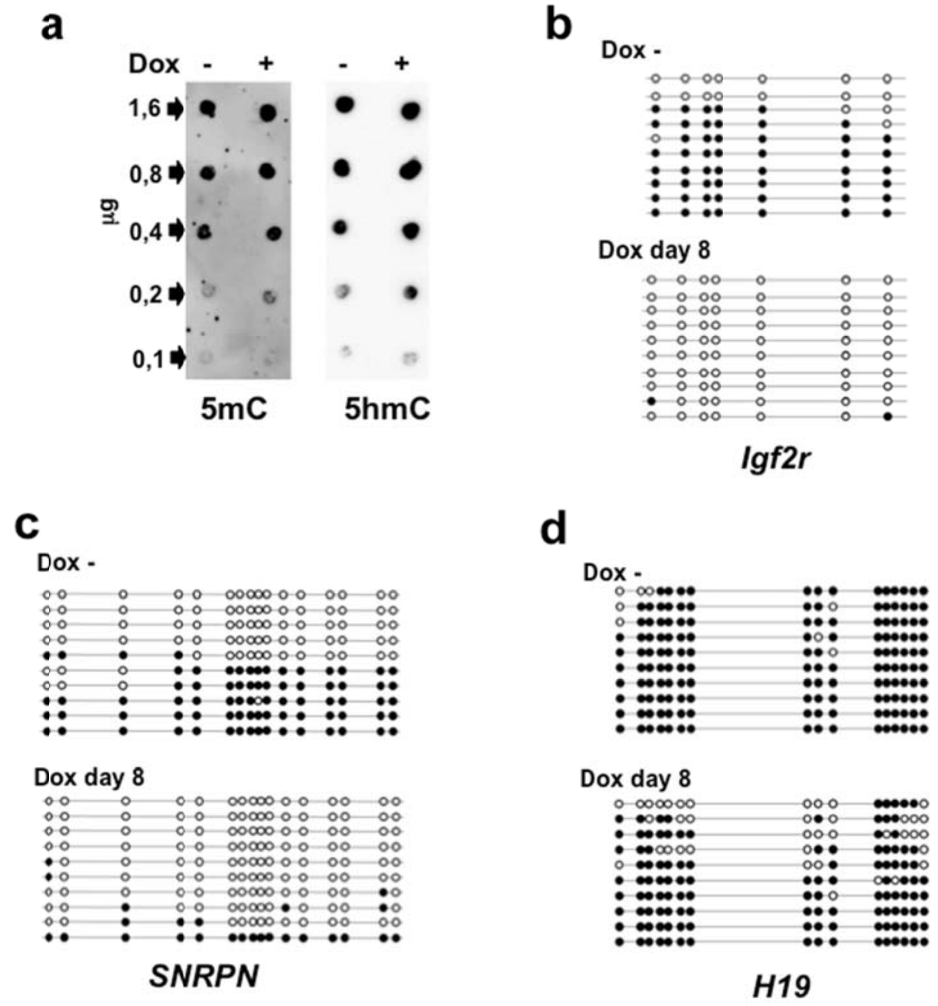

Supplementary Figure 6 Examination of changes in the DNA methylation status of *Max* expression-ablated ESCs. (**a**) Comparison of 5mC and 5hmC levels in genomic DNAs between Dox-untreated *Max*-null ESCs and those treated with Dox for 8 days by dot blot analyses. (**b**–**d**) Bisulfite sequencing analyses were performed on maternally methylated DMRs of *Snrpn* (**b**) and *Ifg2r* (**c**) genes, and the paternally methylated DMR of the *H19* (**d**) gene with the same genomic DNAs used in **a**.

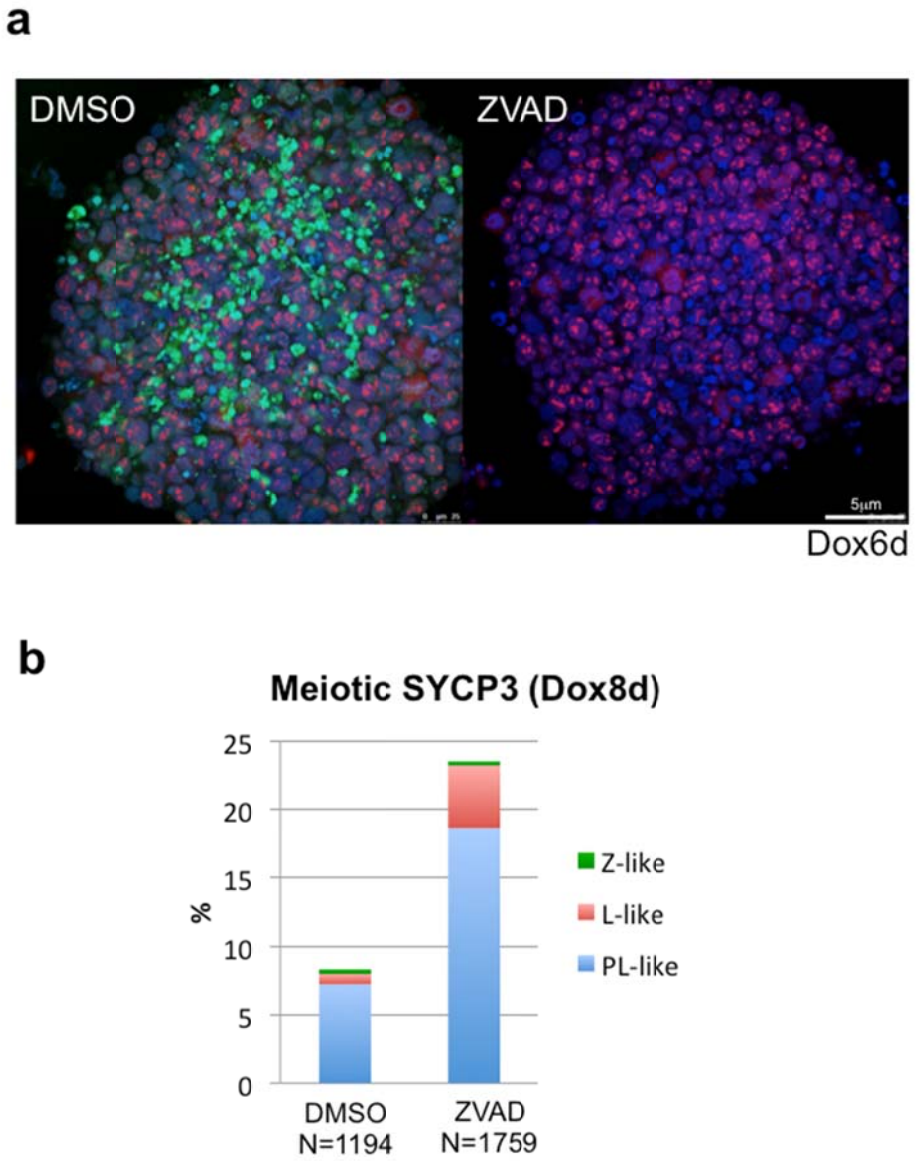

Supplementary Figure 7 Effect of a caspase inhibitor on meiosis-like changes in *Max*-null ESCs. *Max*-null ESCs were treated with Dox and a caspase inhibitor, Z-VAD-FMK, or Dox alone with solvent (DMSO) for 6 days. These cells were then subjected to immunostaining for SYCP3 (pink). Activation levels of caspase-3/7 (green) were assessed using CellEvent Caspase-3/7 green detection reagent from Invitrogen. Cells were counterstained with DAPI. Assignment of the stage of meiosis-like cells derived from *Max*-null ESCs was performed according to the criteria in Supplementary Fig. 3.

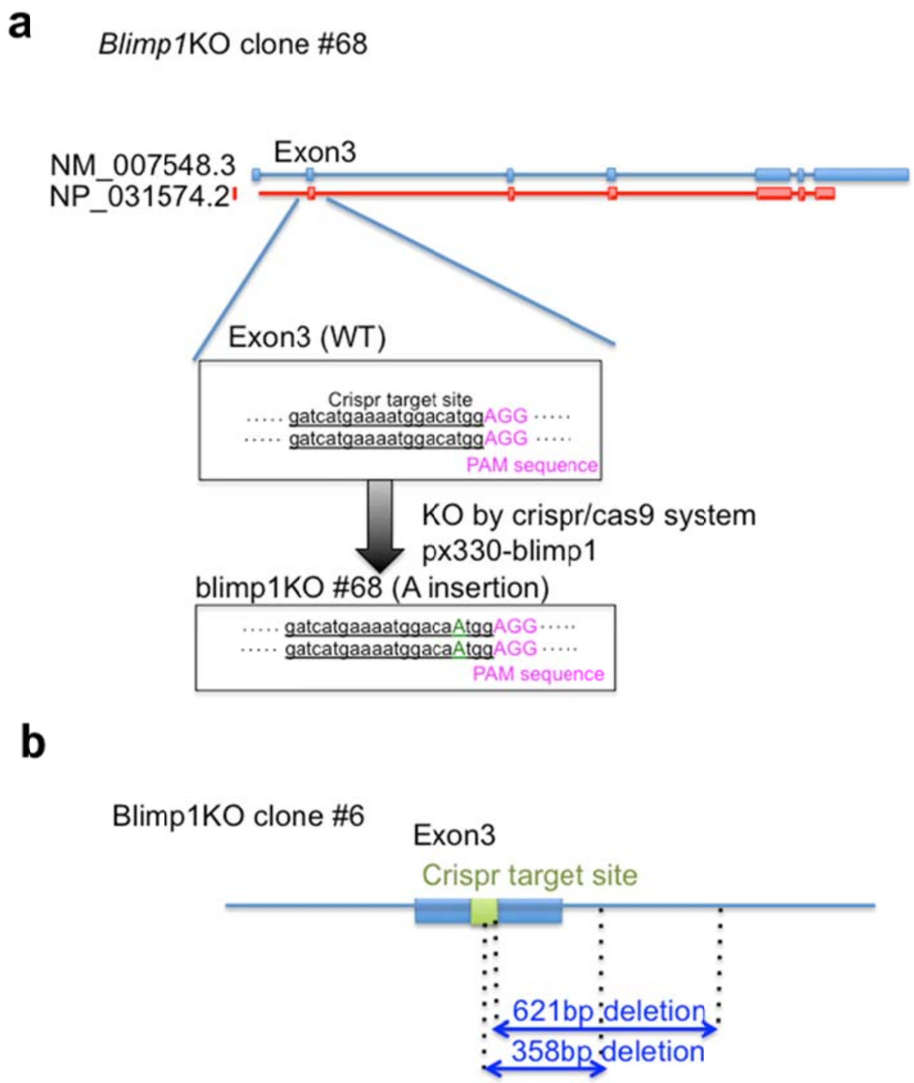

Supplementary Figure 8 Schematic representation of two *Max*-null ESC clones without functional *Blimp1* genes. (**a**) *Blimp1* KO #68 *Max*-null ESCs. Adenine was inserted into the initiating ATG codon in both loci, making the coding sequence out of frame. Homozygosity of the mutation was confirmed by direct sequencing of PCR products without the subcloning step. (**b**) *Blimp1* KO #6 *Max*-null ESCs. Regions encompassing parts of exon 3 and intron 3 of the *Blimp1* gene were deleted in both loci as indicated. Therefore, functional BLIMP1 protein would not be produced because of an artificial stop codon at the immediate downstream portions of both strands.

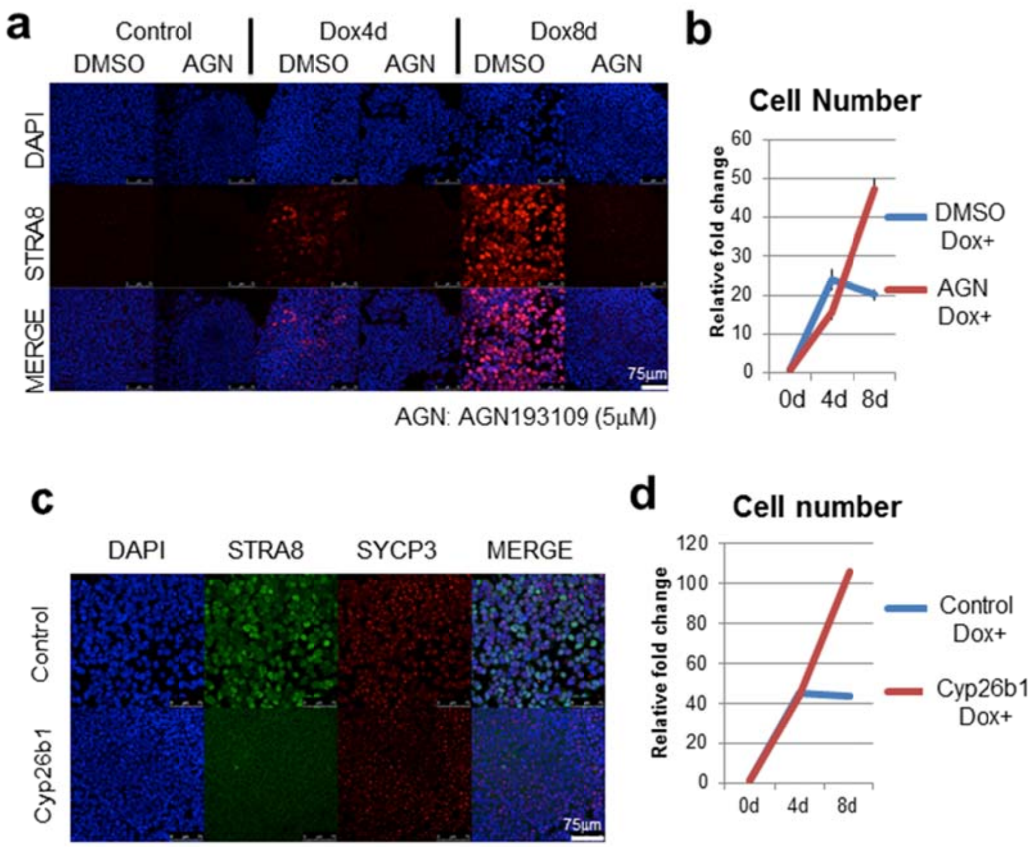

Supplementary Figure 9 Removal of functional RA results in elimination of meiosis-related changes in Dox-treated *Max*-null ESCs. (**a**) Dox-treated and untreated *Max*-null ESCs cultured in the presence or absence of AGN for the indicated days were subjected to immunostaining with an antibody against STRA8. (**b**) Loss of meiosis-like changes was accompanied by an increase in the recovery of viable cells. Viable cells were counted with respect to Dox-treated *Max*-null ESCs cultured either in the presence or absence of AGN. The number of cells at the starting point was arbitrarily set to one (n=3, means±s.d.). (**c**) Expression of *Cyp26b1* significantly lowered the levels of meiosis-like changes in *Max*-null ESCs. *Max*-null ESCs with stable transfection of a *Cyp26b1* expression vector under the control of the constitutive β-actin promoter or an empty vector were treated with Dox for 8 days and then co-immunostained with antibodies against STRA8 and SYCP3. (**d**) Expression of *Cyp26b1* led to an increase in the recovery of viable Dox-treated *Max*-null ESCs. Numbers of viable *Max*-null ESCs with or without expression of *Cyp26b1* were counted at the indicated days during Dox treatment. The number of cells at the starting point was arbitrarily set to one.

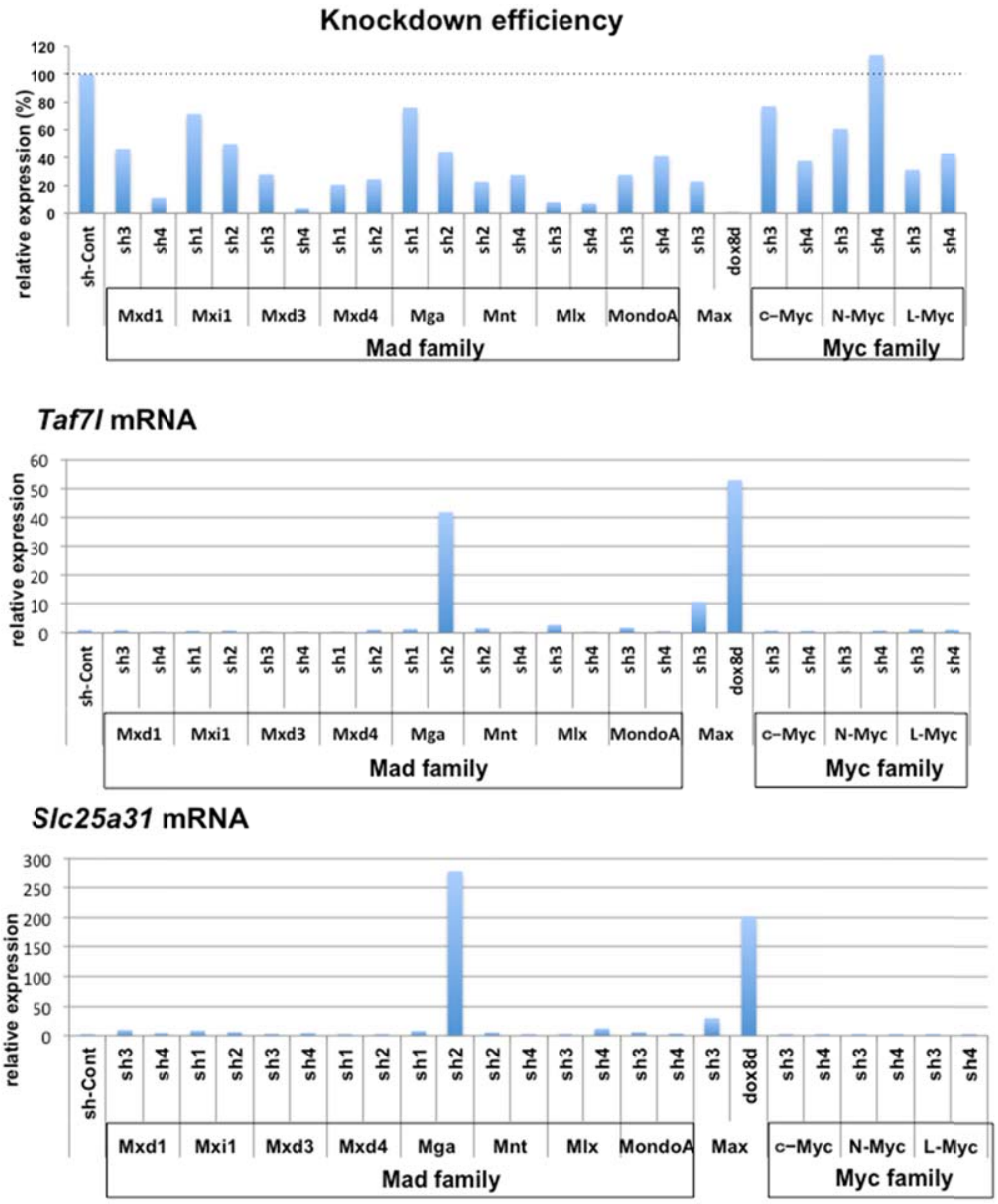

Supplementary Figure 10 Relationship between knockdown of Myc superfamily genes and induction of meiotic gene expression. (**a**) Lentivirus-mediated knockdown of individual genes was conducted as shown in Fig. 7 with the sequences shown in Supplementary Table 1. RNA levels obtained with the scrambled sequence for knockdown were arbitrarily set to 100%. (**b**,**c**) Quantitative PCRs were conducted with cDNAs from RNA of Dox-untreated *Max*-null ESCs in which expression of either one of the *Myc* superfamily genes had been down-regulated by lentivirus-mediated knockdown to determine the expression levels of *Taf7l* (**b**) and *Slc25a31* (**c**).

Max cKO ESCs

Relative expression of Max null ESCs (Dox- =1)

	Gene Name	Dox1d	Dox2d	Dox3d	Dox4d	Dox6d	Dox8d
	Max	-	-	-	-	-	-
Mad family	*Mxd1*	0.94	0.92	0.86	0.76	1.03	1.01
	Mxi1	0.92	1.08	0.76	0.90	0.63	0.64
	Mxd3	0.87	0.64	0.66	0.55	0.48	0.54
	Mxd4	0.92	0.85	0.97	0.99	1.54	2.36
	Mga	3.22	3.16	4.30	4.45	1.66	0.80
	Mlx	0.76	0.72	0.79	0.58	0.49	0.63
	MondoA	0.82	0.80	0.82	0.86	1.78	2.03
	Mnt	1.33	1.01	1.37	1.34	0.67	0.72
Myc family	*Myc*	1.45	0.50	1.78	1.68	1.06	1.12
	Mycn	1.34	0.96	1.90	2.09	1.76	0.42
	Mycl	0.90	0.91	1.06	1.17	1.31	1.78
PRC1	*L3mbtl2*	0.66	0.75	0.79	0.72	0.76	0.74
	Rybp	1.87	1.31	1.46	1.55	1.36	1.35
	Rnf2	1.48	1.67	1.84	2.27	1.30	1.10
	E2F6	1.23	0.70	0.90	0.78	1.15	1.02

0.02 50

Supplementary Figure 11 Effect of *Max* expression ablation in ESCs on expression of other *Myc* superfamily genes and genes encoding one of the components of the PRC1.6 complex. Expression data of *Myc* superfamily genes and genes encoding PRC1.6 complex components during Dox treatment of *Max*-null ESCs were extracted from DNA microarray data (GSE27881).

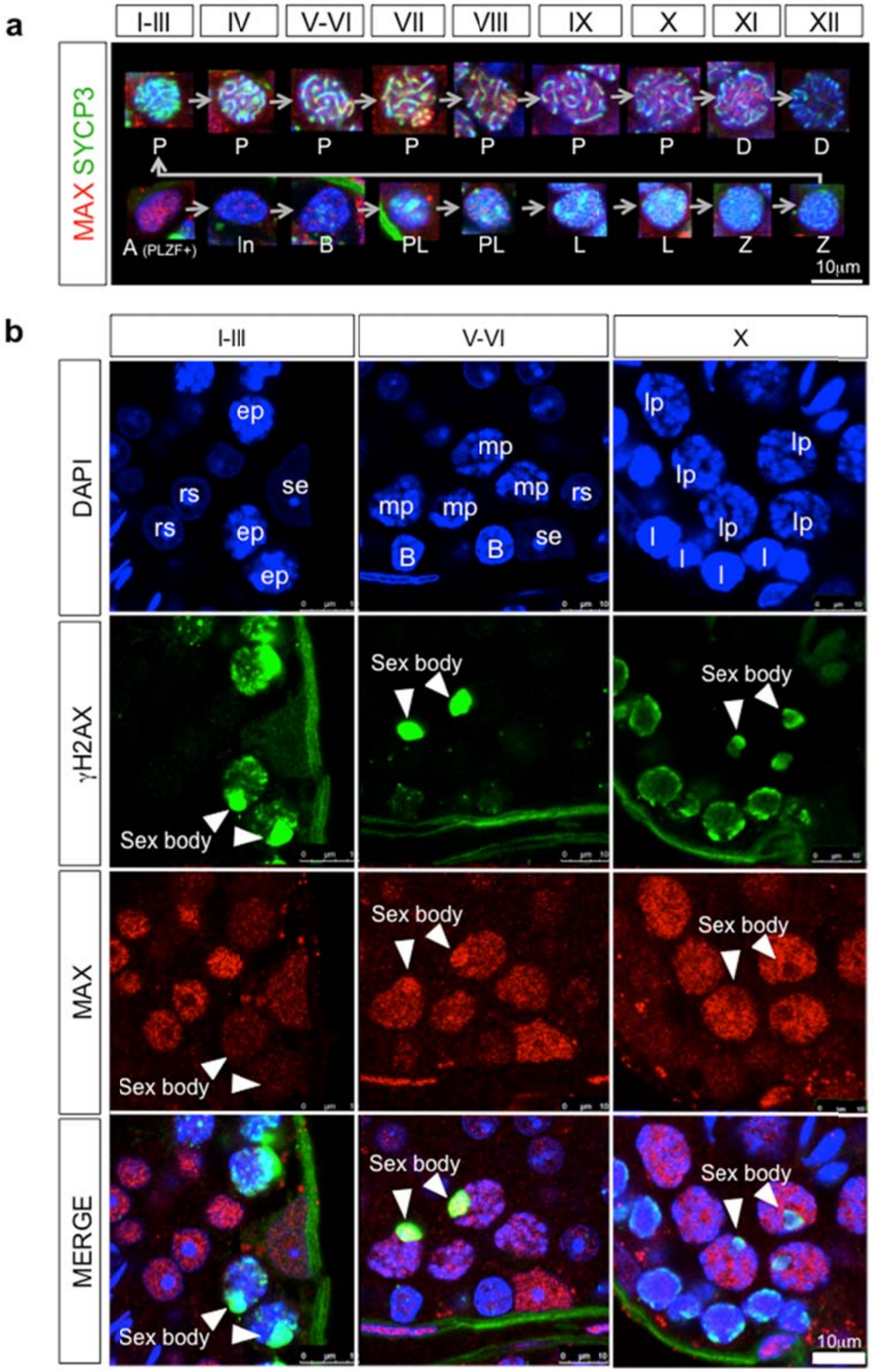

a
I-III
IV
V-VI
VII
VIII
IX
X
XI
XII
MAX SYCP3
P
P
P
P
P
P
P
D
D
A (PLZF+)
In
B
PL
PL
L
L
Z
Z
10μm
b
I-III
V-VI
X
DAPI
ep
rs
se
rs
ep
ep
mp
mp
mp
mp
rs
B
B
se
lp
lp
lp
lp
lp
l
l
l
l
γH2AX
Sex body
Sex body
Sex body
MAX
Sex body
Sex body
Sex body
MERGE
Sex body
Sex body
Sex body
10μm

Supplementary Figure 12 Comprehensive examination of MAX protein expression levels during the cycle of the seminiferous epithelium in testes. (**a**) Immunocytochemical analyses of the seminiferous epithelium were conducted with an antibody against MAX. Stages of the spermatogenic cycle were determined by the expression pattern of co-immunostained SYCP3 and the nuclear shape of spermatids visualized by DAPI staining. A-type, B-type and intermediate spermatogonia are denoted as A, B, and In, respectively, while PL, L, Z, P and D represent preleptotene, leptotene, zygotene, pachytene and diplotene, respectively. (**b**) Immunostaining of MAX in cells at the pachytene stage of meiotic cell division. Cells at the pachytene stage were identified by strong immunostaining for γH2AX in the sex body, a characteristic structure observed at this stage. Germ cells at the pachytene stage in I, VII and X stages of the seminiferous epithelium cycle were designated as cells at early (ep), middle (mp) and late (lp) pachytene stages, respectively. rs, round spermatid.

a

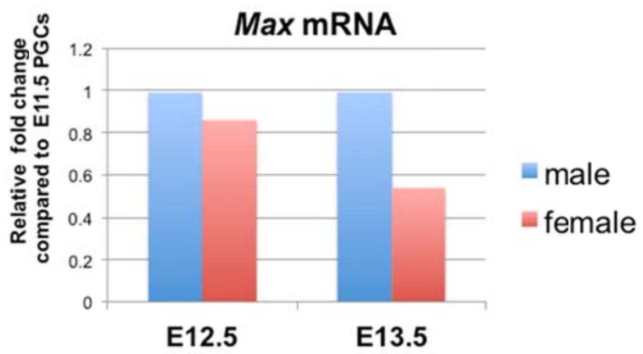

b

PGCs	Gene Name	Relative expression of PGCs (E9.5 PGCs=1)					
		E10.5	E11.5	E12.5 Male	E12.5 female	E13.5 Male	E13.5 female
Meiosis	*Stra8*	1.81	0.81	1.03	46.51	2.70	299.97
	Dazl	1.99	12.99	37.41	39.31	26.27	36.62
	Ddx4	3.15	6.62	9.78	10.71	11.80	13.14
	Slc25a31	4.60	10.34	14.17	16.28	25.34	35.25
	Stk31	3.18	6.67	9.35	12.50	17.80	21.06
	Taf7l	1.16	1.65	1.83	3.89	13.51	62.06
	Tdrd9	1.31	1.38	1.97	2.59	4.16	5.46
	Sycp2	2.67	7.44	9.86	8.15	49.30	44.80
Meiosis & 2C*	*Sycp1*	2.61	4.21	8.03	16.69	49.61	108.37
	Sycp3	1.97	4.91	5.73	8.89	11.95	34.86
	Hormad1	6.88	15.77	18.28	30.24	30.74	53.62
	Mov10l1	4.66	12.59	22.75	28.40	28.66	44.51
	Tex101	2.83	7.27	6.53	15.49	12.38	103.58
	Tex11	2.22	2.14	5.81	5.06	16.45	9.58
	Tex15	2.37	3.87	3.74	6.20	6.20	12.36
2C	*Zscan4*	1.01	1.36	1.02	0.65	1.27	1.24
	Dub1	0.41	0.33	0.42	0.36	0.40	0.46
	Eif1a	0.60	0.31	1.03	0.37	0.47	0.68
	Gm4340	0.87	0.84	0.85	0.85	0.53	0.68
	Gm8300	1.21	0.96	0.76	1.20	1.10	0.97
	Tcstv3	0.62	0.86	0.70	0.78	0.72	1.04
	Zfp352	0.87	1.47	1.00	0.43	1.08	0.53

0.02 50

Supplementary Figure 13 Comparison of expression of meiotic and two-cell signature genes between male and female PGCs. (**a**) Expression signal values of *Max* in male and female PGCs at 12.5 and 13.5 dpc. Expression values of *Max* in PGCs were extracted from DNA microarray data deposited under accession number GSE40412. (**b**) Expression signal values of germline-specific genes and/or two-cell signature genes in PGCs from 13.5 dpc embryos. Expression values of germline-specific genes and/or two-cell signature genes shown in Supplementary Fig. 1 were extracted from the above DNA microarray data of male and female PGCs.

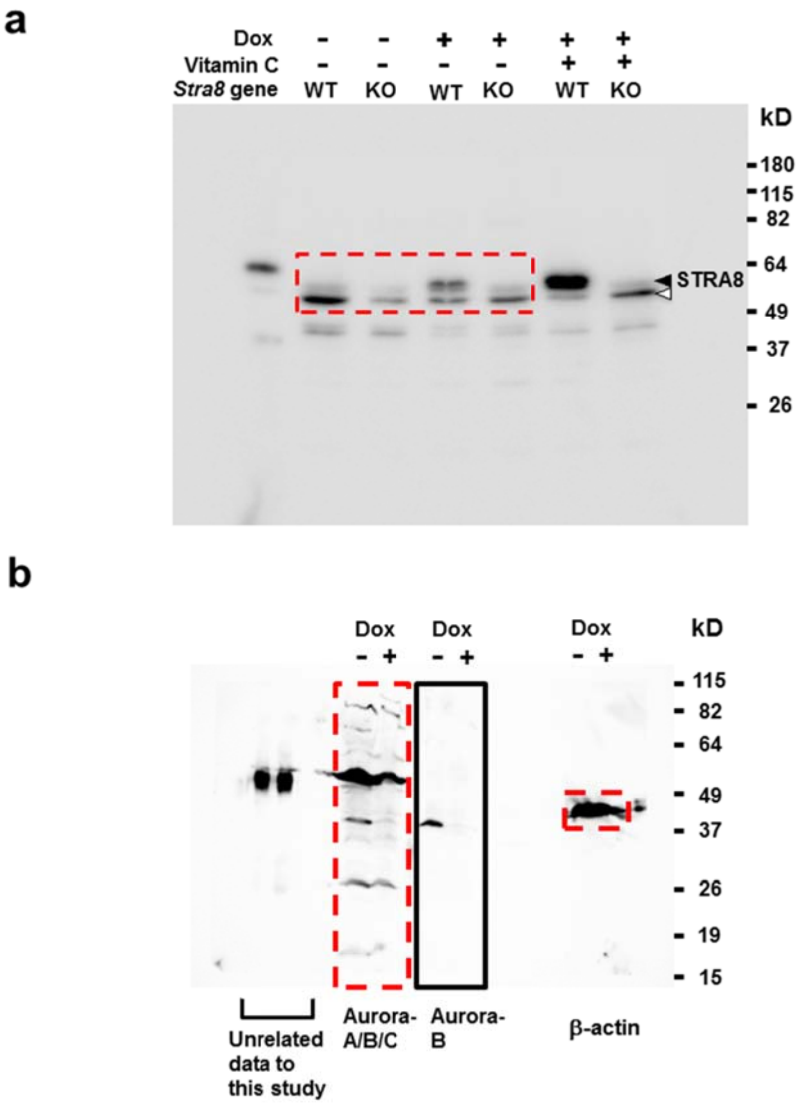

Supplementary Figure 14 Unprocessed western blots. (**a**) Uncropped version of western blot scans in which red dashed lines delineate the region used as Figure 4c. The sold and open triangles indicate specific and non-specific bands reactive to anti-STRA8 antibody as in Figure 4c. kD, kilodalton. (**b**) Uncropped version of western blot scan obtained with an antibody recognising all three kinds of Aurora kinase (AURORA-A/B/C) and internal control β-actin. The regions used for supplementary Figure 5a. are indicated by red dashed lines. The solid rectangle shows western blot scan obtained with the same set of samples (whole cell extracts from Dox-untreated and –treated *Max*-null ESCs) using antibody specific for AURORA-B for a precise assessment of three different kinds of Aurora kinases (A/B/C) in SDS-polyacrylamide gel.

Supplementary Table 1

Sequence used for lentivirus-mediated knockdown

Max sh3	5'-CCGGGAGCAACCGAGGTTTCAATTTCAAGAGAATTGAAACCTCGGTTGCTCTTTTTT-3'
Mxdl sh3	5'-CCGGAAGGACAGAGATGCCTTCAAATTCAAGAGATTTGAAGGCATCTCTGTCCTTTTTTTT-3'
Mxdl sh4	5'-CCGGTAGACATACTATCCTAGTTAATTCAAGAGATTAACTAGGATAGTATGTCTATTTTTTT-3'
Mxi1 sh1	5'-CCGGTAGACATACTATCCTAGTTAATTCAAGAGATTAACTAGGATAGTATGTCTATTTTTTT-3'
Mxi1 sh2	5'-CCGGTTCAGAAATGTTATCTTCTAATTCAAGAGATTAGAAGATAACATTTCTGAATTTTTTT-3'
Mxd3 sh3	5'-CCGGCCCAGGGAATTTCATGTAGCATTCAAGAGATGCTACATGAAATTCCCTGGGTTTTTT-3'
Mxd3 sh4	5'-CCGGTTGACTGTACCCGATACACTATTCAAGAGATAGTGTATCGGGTACAGTCAATTTTTTT-3'
Mxd4 sh1	5'-CCGGCAGCGGGAACATCGCTTCCTATTCAAGAGATAGGAAGCGATGTTCCCGCTGTTTTTT-3'
Mxd4 sh2	5'-CCGGTTCCAGAAGTATTAAATATCATTCAAGAGATGATATTTAATACTTCTGGAATTTTTT-3'
Mga sh1	5'-CCGGCACAATAAAGTTGATAATTTATTCAAGAGATAAATTATCAACTTTATTGTGTTTTTT-3'
Mga sh2	5'-CCGGCAGGCTTAGATTCAAATTTAATTCAAGAGATTAAATTTGAATCTAAGCCTGTTTTTT-3'
Mnt sh2	5'-CCGGCCCTGTCGCACCAGCAAGTAATTCAAGAGATTACTTGCTGGTGCGACAGGGTTTTTT-3'
Mnt sh4	5'-CCGGATGAGTCCCAACCAACAATAATTCAAGAGATTATTGTTGGTTGGGACTCATTTTTTT-3'
Mlx sh3	5'-CCGGCACAATGTTGAACCTACCCAATTCAAGAGATTGGGTAGGTTCAACATTGTGTTTTTT-3'
Mlx sh4	5'-CCGGCACAGCCTTGAAGATAATGAATTCAAGAGATTCATTATCTTCAAGGCTGTGTTTTTT-3'
MondA sh3	5'-CCGGCACAGACATGCTCATGTCAGATTCAAGAGATCTGACATGAGCATGTCTGTGTTTTTT-3'
MondA sh4	5'-CCGGCAGGGCCTTGTTATCACAGCATTCAAGAGATGCTGTGATAACAAGGCCCTGTTTTTT-3'
c-Myc sh3	5'-CCGGACTTCACCAACAGGAACTATGTTCAAGAGACATAGTTCCTGTTGGTGAAGTTTTTTT-3'
c-Myc sh4	5'-CCGGGAATTTCTATCACCAGCAATTCAAGAGATTGCTGGTGATAGAAATTCTTTTTT-3'
N-Myc sh3	5'-CCGGCAGTTGCTAAAGAAGATCGAATTCAAGAGATTCGATCTTCTTTAGCAACTGTTTTTT-3'
N-Myc sh4	5'-CCGGGAAGAGACGTTCCTCCTCTAATTCAAGAGATTAGAGGAGGAACGTCTCTTCTTTTTT-3'
L-Myc sh3	5'-CCGGGGTGAAGAGATTGACGTGGTTCAAGAGACCACGTCAATCTCTTCACCTTTTTT-3'
L-Myc sh4	5'-CCGGGCACTTCCATATCTCTATCTTCAAGAGAGATAGAGATATGGAAGTGCTTTTTT-3'
L3mbtl2 sh1	5'-CCGGCCGATATGAAGGCTTTGAAATTCAAGAGATTTCAAAGCCTTCATATCGGTTTTTT-3'
L3mbtl2 sh2	5'-CCGGGGCCATTGACCCTCTGAATCTTTCAAGAGAAGATTCAGAGGGTCAATGGCCTTTTTT-3'
